# Mechanochemical‐Triggered Confined Coordination of Iron‐Biomass Composites for Efficient Cr(VI) Reduction Under Circumneutral pH Via Accelerated Electron Extraction

**DOI:** 10.1002/advs.202417368

**Published:** 2025-02-24

**Authors:** Yue Wang, Hongyu Li, Hao Liu, Chengsi Hou, Zhengwei Zhou, Shuai Peng, Zuofeng Chen, Zhendong Lei, Deli Wu

**Affiliations:** ^1^ State Key Laboratory of Pollution Control and Resources Reuse College of Environmental Science & Engineering Tongji University Shanghai 200092 P. R. China; ^2^ School of Chemical Science and Engineering Tongji University Shanghai 200092 P. R. China; ^3^ Shanghai Institute of Pollution Control and Ecological Security Shanghai 200092 P. R. China

**Keywords:** circumneutral pH, confined coordination reaction, Cr(VI) detoxification and tethering, green synthesis, scale‐up extended application

## Abstract

Green and eco‐friendly iron‐based materials for efficient Cr(VI) removal have attracted considerable interest, but challenges related to narrow working pH ranges and iron utilization efficiency still remain. Herein, inspired by the hot‐spot effect‐triggered confined coordination strategy, a biomass‐confined iron‐based reductant (CMC‐GTB/Fe^bm^) is designed for Cr(VI) reduction and detoxification. Electron enrichment and confinement on biomass carriers are achieved through electron transfer mediated by coordination interactions between anchored iron species and biomass. Thus, the CMC‐GTB/Fe^bm^ achieved 99% Cr(VI) reduction at circumneutral pH (5–9), with a maximum removal capacity of 180 mg g^−1^. Under iron dosing close to the stoichiometric ratio (Fe/Cr = 3/1), the Cr(VI) removal kinetics and efficiency of CMC‐GTB/Fe^bm^ are 53.2–870.5 and 5.5–48.8 times higher than those of micro‐ or nano‐zero‐valent iron (ZVI), respectively. Mechanistic analyses revealed that confined electron transfer is facilitated by coordination interactions between biomass and anchored iron species, which enhanced Cr(VI) reduction. Moreover, biomass‐tethered reduced Cr(III) is stabilized by electrostatic adsorption and biomass‐Cr(III) coordination, which ultimately detoxifies the phytotoxicity of Cr(VI). The conversion of this strategy to kilogram‐scale production and the simulated Cr(VI) removal in real water matrices are confirmed. This study provides a basis for the controlled design and industrial application of environmentally friendly iron‐based reductants.

## Graphical abstract

This study delineates a mechanochemical‐triggered confined metal‐biomass coordination strategy that demonstrates strong potential for industrialization. The prepared CMC‐GTB/Fe^bm^ material achieved efficient Cr(VI) reduction and detoxification under circumneutral pH (5–9). The confined Fe(II)/Fe(III) cycle accelerated electron extraction, continuously capturing electrons from the biomass for Cr(VI) reduction, and ultimately reduced the iron dosage to a theoretical value (Fe/Cr = 3/1).

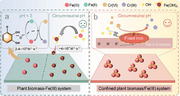

## 1 Introduction

As an important metallic element, chromium (Cr) finds extensive application in industrial processes such as metallurgy, electroplating, printing and dyeing, and leather tanning.^[^ ^1^ ^]^ The natural composition of the Cr element includes hexavalent chromium (Cr(VI)) and trivalent chromium (Cr(III)).^[^ ^2^ ^]^ Among them, Cr(VI) is highly toxic and carcinogenic and is classified as a class I human carcinogen by the US Environmental Protection Agency (USEPA, 1998).^[^ ^3^ ^]^ In addition, Cr(VI) predominantly occurs as anions such as Cr_2_O_7_^2−^, HCrO_4_^−^, and CrO_4_^2−^, which are associated with a high tendency to migrate in environmental media.^[^ ^4^ ^]^ Yang et al. assessed the average concentration of Cr in industrial area soils as being 0.5 times higher than the soil quality standard of China (GB15618‐1995), with 10.7% even exceeding 200 mg kg^−1^.^[^ ^5^ ^]^ On the other hand, Cr(III) functions as an essential trace element for plant and animal growth and becomes immobilized by forming hydroxide precipitates as the pH rises.^[^ ^6^ ^]^ Thus, in comparison with adsorption, reduction, and precipitation are the critical strategies to achieve efficient Cr(VI) removal and detoxification, such as chemical reduction and photocatalytic reduction.^[^ ^7^ ^]^

Iron is the fourth abundant metal element in the earth's crust and critical for the development of life.^[^ ^8^ ^]^ Iron‐based reductants, which exhibit environmental amenity and strong reducibility, are expected to be reliable reductants for Cr(VI) removal, such as nano and micro zero‐valent iron (ZVI), green synthesized iron nanoparticles, Fe(II)‐bearing minerals, and green rust.^[^ ^9^ ^]^ However, the co‐existing oxidizing substances diminish the electron utilization efficiency from iron sources (e.g., Fe^0^, Fe(II)), causing a several‐fold increase in the stoichiometric ratio of iron dosing for effective Cr(VI) reduction.^[^ ^8^, ^10^ ^]^ Another nearly unsolved challenge is the large‐scale production of such materials, which is crucial for practical applications. Most conventional methods rely on liquid‐phase reduction for iron nanoparticle preparation, requiring tight control of reaction pH, temperature, and gas atmosphere.^[^ ^10^, ^11^ ^]^ These usually entail costly and time‐consuming processing, hindering scale‐up for industrialization.^[^ ^12^ ^]^ Hence, 1) enhancing the utilization efficiency of iron species and 2) establishing a straightforward protocol for iron reductant production are the major challenges for sustainable Cr(VI) reduction.

Plant biomass is abundantly available on earth and considered as an eco‐ and environment‐friendly green reductant. Various plant extracts (mainly polyphenols) are proven to reduce Fe(III) by electron transfer in the absence of exogenous chemical reductants, e.g., tea, coffee, fruits, and wines, etc,^[^ ^13^ ^]^ and this advantage was reported for Cr(VI) removal. However, many studies revealed the Cr(VI) removal by plant extract‐synthesized Fe nanoparticles but with conventional results,^[^ ^14^ ^]^ with remaining limitations in iron utilization efficiency. Although stoichiometric removal of Cr(VI) by extracted organic matter‐Fe(III) complexes has been partially reported in the literature, it faces the limitation of acidic reaction (pH < 3).^[^ ^15^ ^]^ The main challenge is that the biomass lacks sufficient binding of iron ions and mitigation of iron precipitation arising from pH changes, as the reaction rate of Fe(III) precipitation (≈4 × 10^7^ M^−1^·s^−1^) > Fe(III)‐polyphenols (2–4 × 10^3^ M^−1^·s^−1^) > Cr(VI)‐polyphenols (< 1 M^−1^·s^−1^).^[^ ^16^ ^]^ Consequently, there is still an enormous challenge to improve the reactivity and pH tolerance of biomass‐iron systems.

**Scheme 1:**
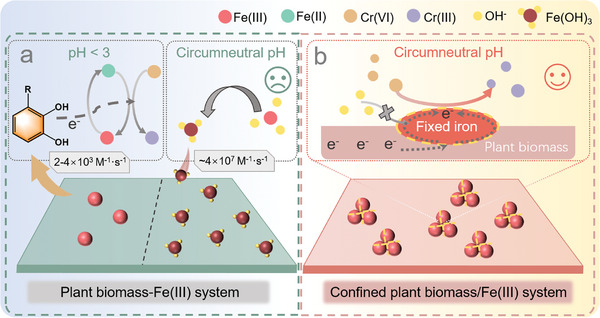
Plant biomass‐confined iron species‐mediated electron confinement. a) Plant biomass‐Fe(III) system without confinement. b) Confined iron species system anchored on plant biomass.

Generally, surface reactions strongly mediate the reductive remediation of contaminants by iron‐based materials. Interfacial confined chemical reactions limit the reacting species within a confined space or carrier, thereby enhancing the selectivity and efficiency of the reactions.^[^ ^17^ ^]^ Therefore, achieving iron species‐confinement thus the electron extraction and confinement at the reaction interface to inhibit the diffusion of iron species and interference from co‐existing oxidizing substances, is expected to enable stoichiometric Cr(VI) removal (**Scheme** **1**). Dry mechanochemistry initiates chemical reactions through the application of mechanical force, which can induce the formation of hot spots and enhance chemical reactions on the surface, aligning with the principles of green chemistry.^[^ ^18^ ^]^ Hence, in this study, we developed a mechanochemical‐triggered confined biomass‐iron coordination strategy. This strategy utilizes click chemical reactions in collisional microregions during ball milling and produces biomass‐iron composites with preferable stoichiometric Cr(VI) removal performance at circumneutral pH. The confined effect resulting from this strategy was confirmed by elemental mapping and iron phase identification. The removal efficiency and resistance of the as‐prepared biomass/iron complexes for Cr(VI) removal under anaerobic conditions were investigated. Concomitantly, the mechanisms of biomass/iron reduction and tethered fixation of Cr(VI) are scrutinized. Finally, the kilo‐scale production potential of the mechanochemical‐triggered confined coordination strategy was demonstrated.

## 2 Results and Discussion

### 2.1 Formation of Confined Biomass/iron Composites

**Figure 1:**
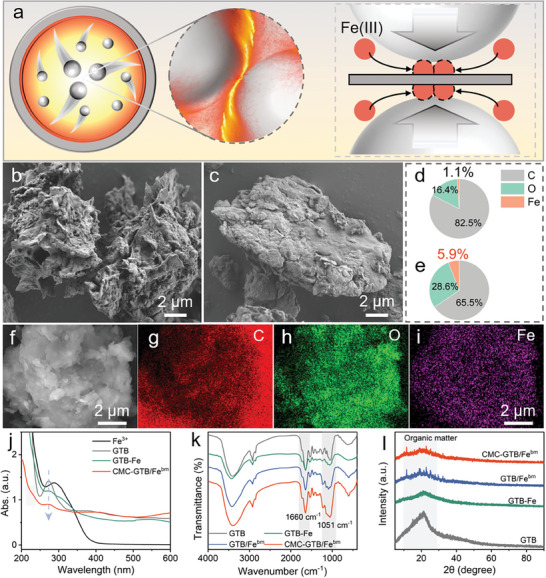
Mechanochemical‐triggered confined coordination strategy toward iron‐biomass composites. a) Schematic illustration of mechanochemical‐triggered confined coordination of biomass‐iron via “hot spot” effect. SEM images of (b) biomass‐iron isolated from a physically mixed solution of tea biomass and iron salt and (c) CMC‐GTB/Fe^bm^ composite. Semi‐quantification of elemental C, O, and Fe on the surface of (d) tea biomass‐Fe and (e) CMC‐GTB/Fe^bm^ materials. f–i) The EDS‐mapping of CMC‐GTB/Fe^bm^ composite. j) FTIR spectra, k) UV–vis spectra, and l) XRD spectra of CMC‐GTB/Fe^bm^ material. Test conditions for UV spectra: 0.1 g L^−1^ dose of materials in aqueous solution.

A solvent‐free, ball milling‐triggered click chemistry reaction was employed to initiate the coordination between biomass and iron salts. This enhanced coordination enabled the confinement of the iron species in the open space (**Figure** **1**a). The CMC‐GTB/Fe^bm^, synthesized through tea biomass and ferric sulfate (Fe_2_(SO_4_)_3_), served as a proof‐of‐concept sample, with carboxymethyl cellulose (CMC) acting as a protecting agent to enhance the stability of this composite system. As shown in Figure S1a,b (Supporting Information), recycled tea biomass rather than tea extract was used for synthesis of this biomass/iron green reductant, unlike previous studies.^[^ ^19^ ^]^ This enhanced coordination reaction benefited from the hot spot effect generated by ball milling, which rapidly elevated the confocal temperature of the collisional micro‐zone,^[^ ^18b^ ^]^ as confirmed by temperature monitoring of the ball milling jars after the reaction (Figure S1c,d, Supporting Information). Thus, efficient synthesis of green reductants under facile operating conditions without external heat transfer or solvent was achieved through this mechanochemically triggered confined coordination reaction of biomass and iron salts.

Scanning electron microscopy (SEM) observations revealed very different surface morphologies of the biomass‐iron and CMC‐GTB/Fe^bm^ materials. Due to the coordination effect of Fe(III) and soluble active species, such as polyphenols,^[^ ^20^ ^]^ the solubilization of biomass was facilitated and a rough surface structure of the biomass‐iron material was formed as shown in Figure 1b. Conversely, the surface structure of the CMC‐GTB/Fe^bm^ composite remained smooth after water dispersion, which was attributed to the hot spot effect‐enhanced coordination reaction confining the iron species on the biomass surface, and thereby inhibiting the solubilization of polyphenols (Figure 1c). The iron loading and its distribution on the material surface were analyzed using energy‐dispersive X‐ray spectroscopy (EDS) mapping. On the surface of the CMC‐GTB/Fe^bm^ material, the loaded iron overlapped with the biomass particles and showed a remarkably strong mapping signal (Figure 1f–i). Compared to this, the mapping signals of iron on the tea biomass‐iron surface were weak and showed a discrete trend, indicating low metal loading (Figure S2, Supporting Information). Semi‐quantitative analyses showed (Figure 1d,e) that the coordination caused by this hot spot effect had an extremely strong confining effect on iron species,^[^ ^21^ ^]^ with the elemental Fe content on the CMC‐GTB/Fe^bm^ surface being 5.4 times higher than that of the tea biomass‐iron material. Meanwhile, XPS survey spectra also showed that the CMC‐GTB/Fe^bm^ material exhibits efficient anchoring of iron species, with nearly three times the tethered iron capacity than GTB‐Fe (Figure S3 and Table S1, Supporting Information). In addition, the iron ion release curve indicated that the majority of iron in the GTB‐Fe system was present in the ionic state, whereas iron was anchored in the CMC‐GTB/Fe^bm^ system, with less than 10 mg L^−1^ of iron ions being released (Figure S4, Supporting Information). This was attributed to the hot spot effect‐induced high‐temperature and high‐pressure micro‐zones that promoted the strong coordination of iron species with biomass components in the reaction sites, which ultimately achieved the anchoring of iron species on GTB biomass.^[^ ^18^, ^21^, ^22^ ^]^

Using spectral characterization to reveal the mechanisms of this open‐space confinement effect. UV absorption spectra showed that the absorption peak of Fe(III) disappeared in both the GTB‐Fe (physical mixture of biomass and iron salts) system and the CMC‐GTB/Fe^bm^ system, as shown in Figure 1j. In addition, the absorption peak at 270 nm of tea biomass, which is attributed to the π‐π* transition of the polyphenol A ring, undergoes a drastic decay in the GTB‐Fe and CMC‐GTB/Fe^bm^ systems.^[^ ^23^ ^]^ Compared with the GTB‐Fe system, the CMC‐GTB/Fe^bm^ system showed a broad peak of quinone at 230 nm, being the oxidation product of phenolics through the reduction of Fe(III) to Fe(II) by single electron transfer.^[^ ^15^ ^]^ Regarding the FTIR spectra, the fingerprint bands attributed to polyphenols at 1000–1800 cm^−1^ in the GTB‐Fe, GTB/Fe^bm^, and CMC‐GTB/Fe^bm^ systems were attenuated, whereas the IR bands at 1051 and 1660 cm^−1^ were enhanced, as shown in Figure 1k. The IR band at 1660 cm^−1^ corresponds to C═O of quinone, formed by the oxidation of phenolic hydroxyl groups (Figure S5, Supporting Information), while the IR band at 1051 cm^−1^ is attributed to the C─O group of polyphenols or catechol from tea biomass.^[^ ^24^ ^]^ The formation of obvious crystalline iron species was not observed in the XRD spectra, suggesting that the iron remains in the amorphous form of coordination compounds (Figure 1l). These phenomena confirm the contribution of phenolics in the confinement of Fe species on the surface of tea biomass, providing a basis for the strong reduction of the CMC‐GTB/Fe^bm^ system.

**Figure 2:**
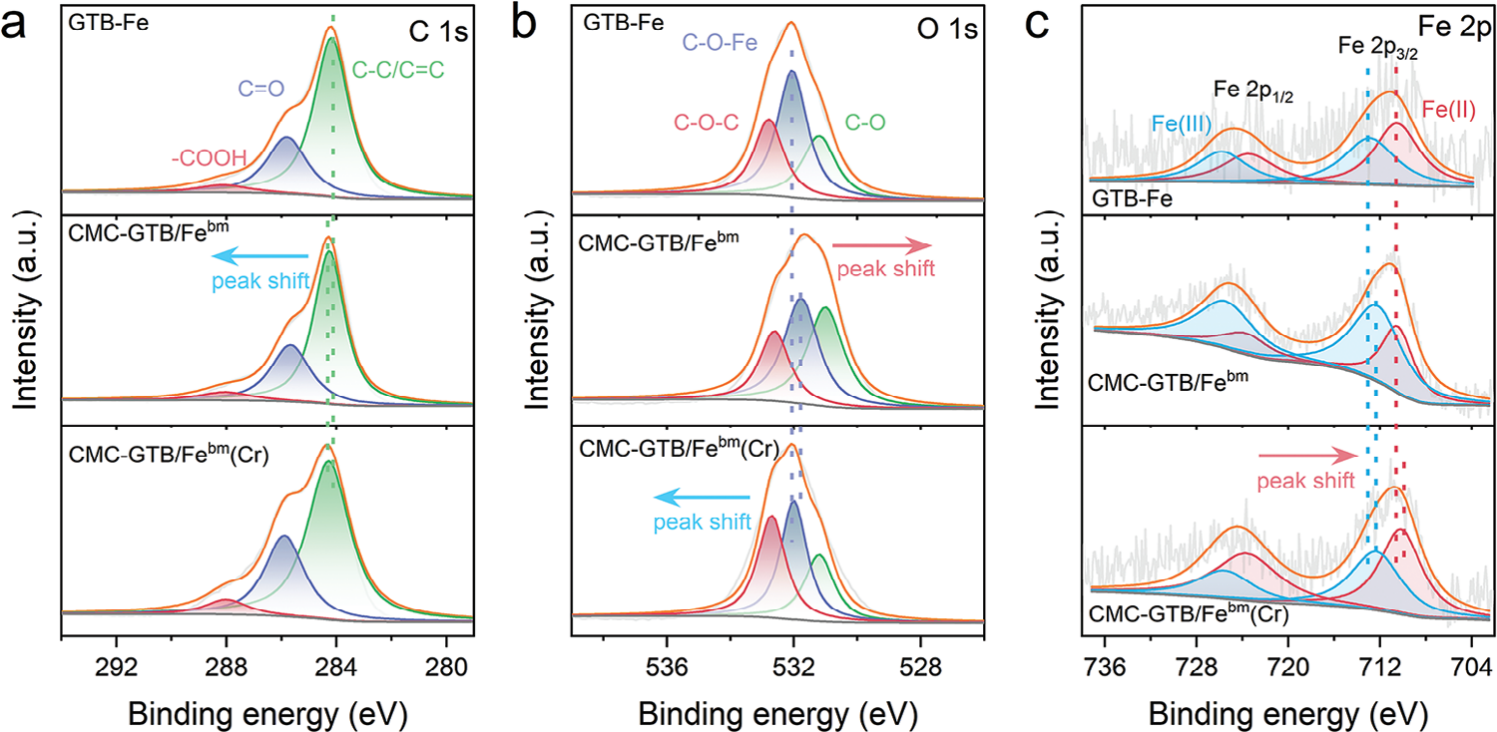
Chemical compositions and charge shift in the active center. High resolution (a) C 1s, b) O 1s, and c) Fe 2p XPS spectra of GTB‐Fe, CMC‐GTB/Fe^bm,^ and CMC‐GTB/Fe^bm^ after Cr(VI) removal (CMC‐GTB/Fe^bm^(Cr)).

To better reveal the active site of the reduction center in CMC‐GTB/Fe^bm^, high‐resolution XPS analysis was employed to investigate the chemical composition of the material, the electronic shifts, and their changes after the reaction. As shown in **Figure** **2**a, the C 1s signal can be deconvoluted into three peaks at 284.2–284.3, 285.7–285.8, and 288.0–288.1 eV, which were attributed to C─C/C═C, C═O, and ─COOH, respectively.^[^ ^25^ ^]^ Meanwhile, as shown in Figure 2b, the O 1s signal can be deconvoluted into three peaks at 531.0–531.2, 531.8–532.1, and 532.6–532.8 eV which were attributed to C─O, C─O─Fe, and C─OH, respectively.^[^ ^26^ ^]^ Compared with GTB‐Fe, the binding energy of C─C/C═C in the CMC‐GTB/Fe^bm^ material is positively shifted, suggesting that the biomass substrate in the material, with the confinement effect, provides a better electron supply to the iron species.^[^ ^27^ ^]^ The negative shift of the binding energy of the C─O─Fe in the CMC‐GTB/Fe^bm^ material is attributed to the stronger interaction between the iron species and the biomass caused by hots pot effect, which facilitates the coordination between biomass and iron, thus increasing the charge density of the C─O─Fe moieties.^[^ ^26^ ^]^ Moreover, the binding energy of C─O─Fe in the CMC‐GTB/Fe^bm^ material shifted positively after Cr(VI) removal, indicating the important role of C─O─Fe moieties in Cr(VI) reduction. Furthermore, the deconvolution of Fe 2p showed four peaks located at 710.2–710.6 and 723.5–723.8 eV, 712.2–712.9 and 725.5–725.8 eV, attributed to Fe(II) and Fe(III), respectively, as shown in Figure 2c. Compared with GTB‐Fe, the Fe 2p_3/2_ deconvolution peaks of Fe(II) and Fe(III) of the CMC‐GTB/Fe^bm^ material are negatively shifted, which further confirms that the electronic shifts due to strong interactions between the iron species and the biomass increase the charge densities of the Fe(II) and Fe(III) moieties.

Therefore, based on the above results, the possible mode of charge transfer at the active center is that the C─C/C═C groups of the active substances such as polyphenols in the biomass donate the electrons; consequently, the C─O─Fe moieties construct a channel for rapid charge transfer between biomass and iron, which ultimately enhances the charge density of the iron active center. This specific charge transfer mode provides the potent reducing activity of CMC‐GTB/Fe^bm^.

### 2.2 Evaluation of Cr(VI) Removal Performance

**Figure 3:**
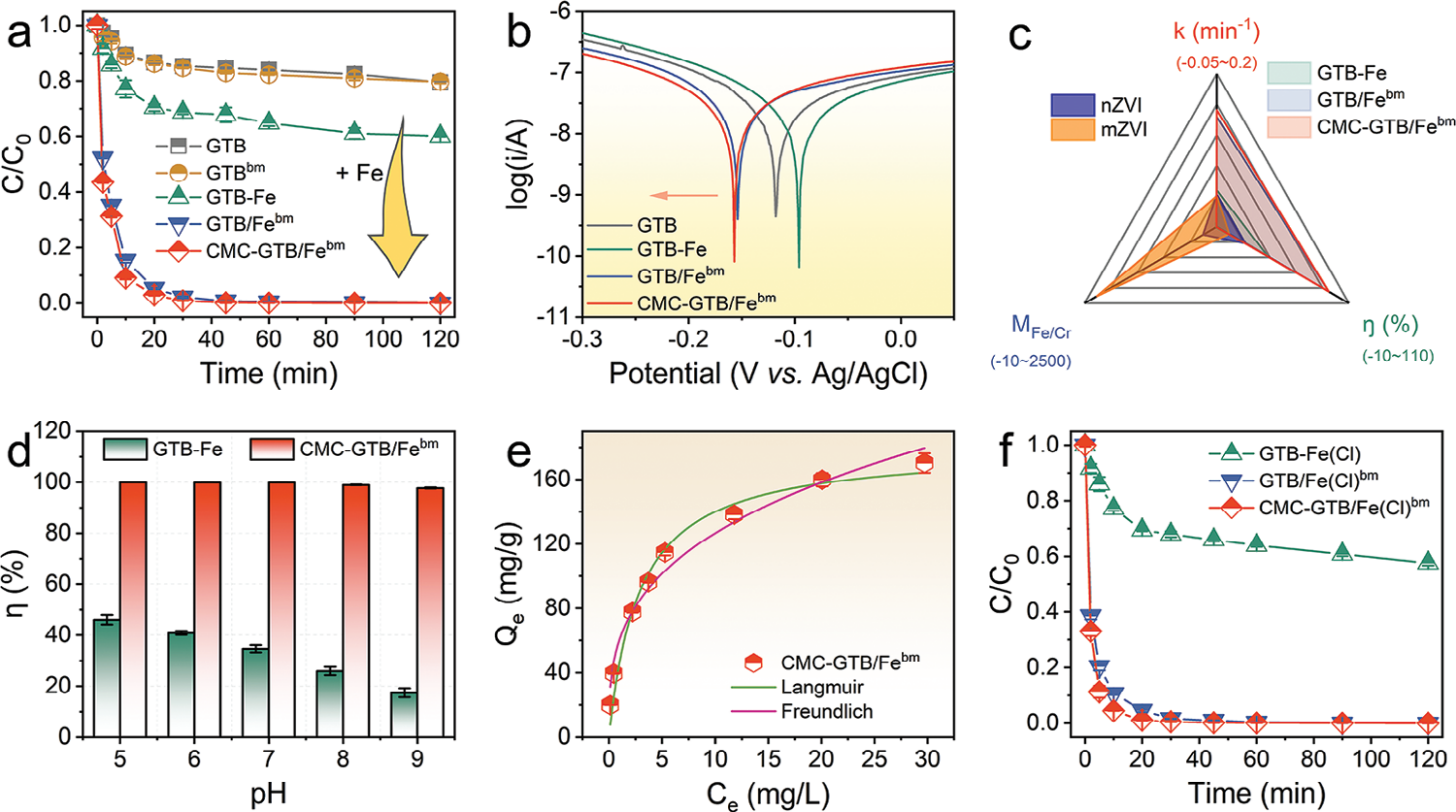
Evaluation of Cr(VI) removal performance by CMC‐GTB/Fe^bm^ composite. a) Cr(VI) removal curves. b) Tafel plots. c) Comparison with zero‐valent iron materials. d) Cr(VI) removal efficiency under different pH. e) Cr(VI) removal isotherm. f) Cr(VI) removal performance of CMC‐GTB/Fe(Cl)^bm^ prepared by ferric chloride (FeCl_3_). Reaction conditions: [Cr(VI)]_0_  =  20 mg L^−1^, [Materials]_0_  =  1.0 g L^−1^, pH_0_   6, T = 273 K.

Cr(VI) removal experiments in aqueous solutions were conducted to assess the reductive activity of the as‐prepared CMC‐GTB/Fe^bm^. As anticipated by the above characterization and analytical results, the GTB/Fe^bm^ and CMC‐GTB/Fe^bm^ composites with confined iron species, exhibited the strongest Cr(VI) removal performance (pH 6). Over 95% of Cr(VI) was removed in only 20 min (**Figure** **3**a; Figure S6, Supporting Information). In contrast, the pristine GTB exhibited minimal Cr(VI) removal performance, even after an ultra‐long reaction period of 48 h (Figure S7, Supporting Information). Notably, this strategy applies likewise to various biomass precursors and can even eliminate the need to use the more reductive ferrous salts as the iron source (Figure S8, Supporting Information). Tafel tests further confirmed the ultra‐negative corrosion potentials of the GTB/Fe^bm^ and CMC‐GTB/Fe^bm^ composites (Figure 3b), suggesting that these materials possess a relatively strong electron‐donating capacity, thereby enhancing Cr(VI) reduction. Meanwhile, CMC‐GTB/Fe^bm^ prepared with different iron salt additions demonstrated excellent Cr(VI) removal performance, surpassing that of GTB‐Fe (Figure S9, Supporting Information). The removal efficiency (η, %), Fe/Cr molar ratio, and kinetic constant (min^−1^) of several iron‐based reduction materials for Cr(VI) removal were comprehensively evaluated, as shown in Figure 3c and Figures S10 and S11 (Supporting Information). The results showed that the Cr(VI) removal kinetics (k) and efficiency (η) of CMC‐GTB/Fe^bm^ were 53.2–870.5 and 5.5–48.8 times higher than those of micron or nano‐zero‐valent iron (ZVI), respectively, at an iron dosage injection close to the stoichiometric ratio (Fe/Cr = 3/1). The results showed that the removal of Cr(VI) by GTB/Fe^bm^ and CMC‐GTB/Fe^bm^ composites had ultra‐fast reaction kinetics, low Fe/Cr ratios, and nearly 100% removal efficiency.

As is well known, the removal of Cr(VI) by iron‐based materials faces a narrow pH range, and the extremely acidic operating pH limits its application.^[^ ^3^, ^14^, ^15^ ^]^ The Cr(VI) removal performance of the CMC‐GTB/Fe^bm^ system was tested in the circumneutral pH range (5–9), while the physical hybrid system of GTB and Fe(III) without confinement effect (GTB‐Fe) was compared, as shown in Figure 3d. These results confirmed that confining iron species on the biomass surface and resisting external pH changes through the pH buffering effect of dissolved plant constituents were effective strategies that maintained the efficient Cr(VI) removal by the CMC‐GTB/Fe^bm^ system.^[^ ^28^ ^]^ Moreover, the adsorption isotherm revealed an ultra‐high capacity of 180 mg g^−1^ for Cr(VI) removal by the CMC‐GTB/Fe^bm^ system at pH 6.0 (Figure 3e; Table S3, Supporting Information), which was even beyond the Cr(VI) removal performance of some iron‐based materials at acidic pH (Table S4, Supporting Information). The CMC‐GTB/Fe(Cl)^bm^ prepared using ferric chloride, another commonly used iron salt, as the precursor also showed ultra‐high reactivity (Figure 3f), confirming the versatility of this strategy for other iron salts.

### 2.3 Cr(VI) Tethering and Phytotoxicity of Reduction Products

**Figure 4:**
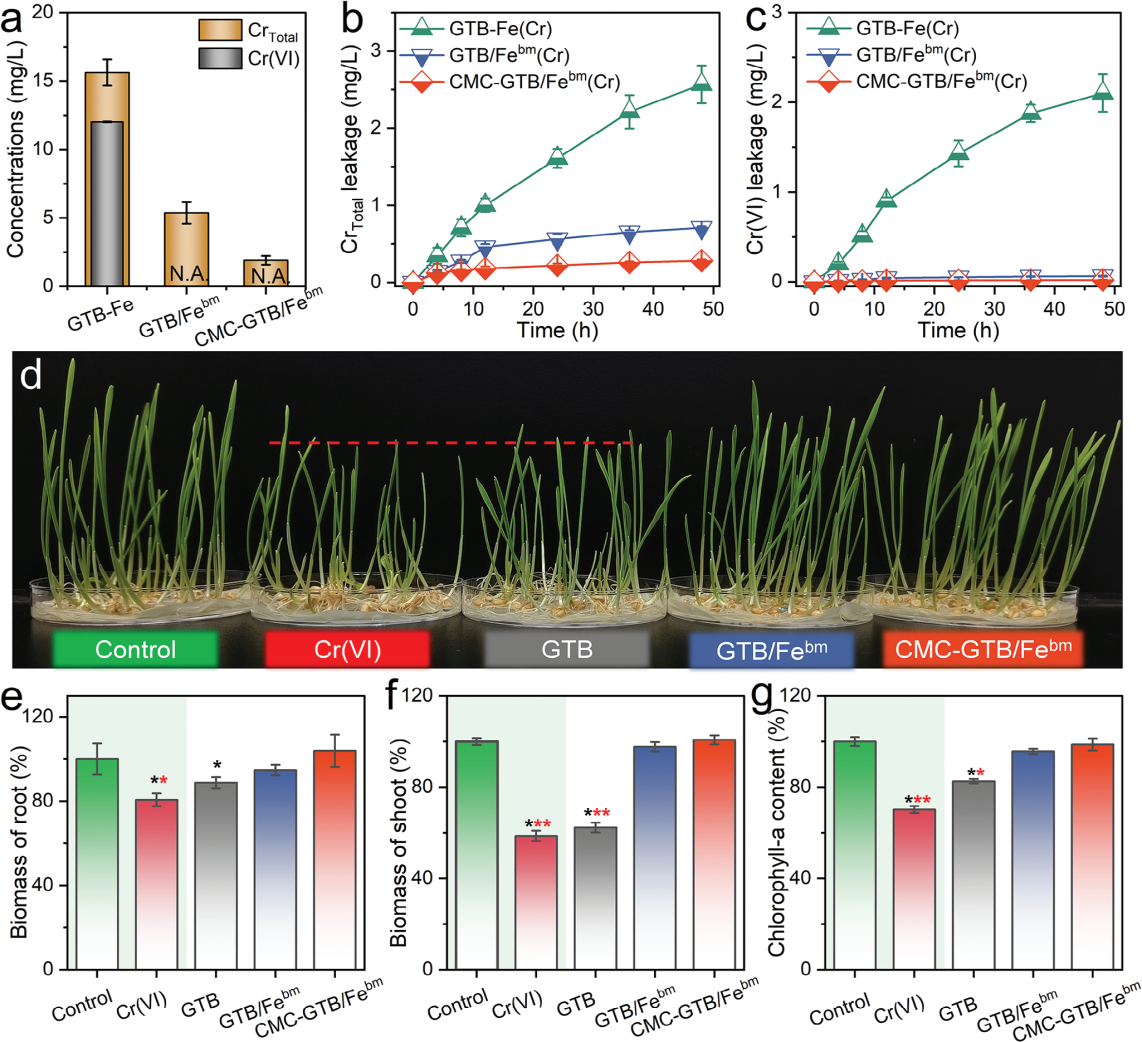
Efficient Cr(VI) reduction and stabilization by CMC‐GTB/Fe^bm^. a) Total Cr remaining in the GTB‐Fe, GTB/Fe^bm,^ and CMC‐GTB/Fe^bm^ systems. b) Total Cr and c) Cr(VI) release from isolated materials after Cr(VI) removal experiments. d) Effects of various systems on wheat (*Triticum aestivum L*) growth (*n* = 6). Effect of post‐reaction solutions on biomass accumulation in wheat (e) roots and (f) shoots. g) Effect of post‐reaction solutions on chlorophyll content in wheat leaves. In the figure, ^*^, ^**^, and ^***^ represent statistical differences with *P* values less than 0.05, 0.01, and 0.001, respectively.

The composition of Cr species in the post‐reaction system was examined to investigate the reduction and tethering capacity of CMC‐GTB/Fe^bm^ for Cr(VI) removal, while the release of Cr species from the material after separation was analyzed. As shown in **Figure** **4**a, Cr(VI) was partially reduced by the unconfined system (GTB‐Fe), and 50% of Cr(VI) remained after the reaction. Comparatively, the confined systems, such as GTB/Fe^bm^, and CMC‐GTB/Fe^bm^, rendered undetectable Cr(VI) after the reaction, suggesting that Cr(VI) was fully reduced in the liquid phase. Furthermore, the Cr release from the separated reductants was tested, and it was found that the unconfined system released up to 3 mg L^−1^ of Cr (mainly Cr(VI)) after the reaction (Figure 4b,c), which could lead to unexpected secondary pollution to the environment. In contrast, GTB/Fe^bm^(Cr) and CMC‐GTB/Fe^bm^(Cr) hardly released Cr(VI), demonstrating an efficient Cr tethering ability. These results confirm the strong reduction and tethering performance of the CMC‐GTB/Fe^bm^ system for Cr(VI) removal.

The efficient detoxification of Cr(VI) by the CMC‐GTB/Fe^bm^ system was further confirmed through plant culture experiments. Wheat (*Triticum aestivum L*), a typical cash crop, was selected as the model plant.^[^ ^29^ ^]^ As shown in Figure S12 (Supporting Information), the germination delaying effect of the solution on wheat was alleviated after CMC‐GTB/Fe^bm^ treatment. In addition, CMC‐GTB/Fe^bm^ treatment attenuated the inhibitory effect of the Cr(VI) solution on the plant height of wheat, as shown in Figure 4d. Investigations on root and shoot biomass showed that the growth of wheat plants was inhibited by limitation in Cr(VI) solution (*p* < 0.001). After the reduction and tethering of Cr(VI) by CMC‐GTB/Fe^bm^, the reacted solution had little inhibitory effect on the biomass of wheat plants (Figure 4e–f). In addition, the photosynthetic pigment chlorophyll content was investigated and the results showed that the CMC‐GTB/Fe^bm^ treatment alleviated the inhibition of chlorophyll synthesis and promoted photosynthesis thereby up‐regulating the accumulation of biomass in the plants.

### 2.4 Mechanism for Superior Cr(VI)‐Reduction and Tethering Capability of Confined Biomass/iron Composites

**Figure 5:**
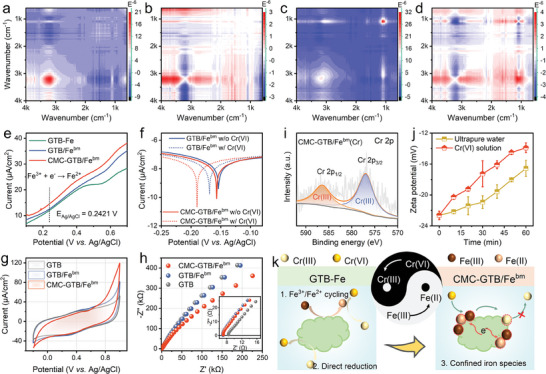
Mechanism of the remarkable Cr(VI) reduction and tethering capacity of CMC‐GTB/Fe^bm^: insights from the functional group and electron transfer. a) Synchronous and b) asynchronous 2DCOS spectra of the unconfined biomass‐iron system (GTB‐Fe) in Cr(VI) solution. c) Synchronous and d) asynchronous 2DCOS spectra of the confined system (CMC‐GTB/Fe^bm^) in Cr(VI) solution. e) Tafel plots of the confined system with and without CMC. f) LSV curves for three biomass and iron compounding systems. g) CV curves and h) Nyquist curves for various materials. i) High‐resolution Cr 2p XPS spectra of CMC‐GTB/Fe^bm^ after Cr(VI) removal. j) Dynamics of zeta potential values of CMC‐GTB/Fe^bm^ in ultrapure water and Cr(VI) solution. k) Mechanism of Cr(VI) removal by CMC‐GTB/Fe^bm^.

2D correlation spectroscopy (2DCOS) was performed to analyze the response of functional groups during Cr(VI) removal. As shown in **Figure** **5**a,b, no functional group response was evidently observed in the synchronous and asynchronous spectra of the GTB‐Fe system, except for the H_2_O peak at 3200 cm^−1^. This was attributed to the precipitation of Fe^3+^ due to pH adjustment and thus limited the reaction of iron with biomass, which ultimately led to inefficient Cr(VI) removal. Compared to GTB‐Fe, iron species are tightly bound to the biomass surface due to hot spot effects, thus enhancing the interaction of biomass components (e.g., polyphenols) with iron salts, and in turn polyphenols act as a capping agent to buffer the pH influence.^[^ ^11^, ^28^ ^]^ Therefore, a strong band of phenolic C─O (1051 cm^−1^) and a weak band of quinone C═O (1660 cm^−1^) were observed in the CMC‐GTB/Fe^bm^ system (Figure 5c,d), suggesting that the confined iron species promoted the aggregation of phenolics from biomass particles toward the reacting solid‐liquid interface (Figure S13, Supporting Information).

High‐resolution XPS spectra of C 1s, O 1s, and Fe 2p revealed rearrangements of charge densities in the C, O, and Fe atomic regions as a result of functional group interactions, indicating a strong tendency for electron transfer (Figure 2).^[^ ^30^ ^]^ Moreover, the increased Fe(II) content in the CMC‐GTB/Fe^bm^ material after the Cr(VI) removal shows the role of Fe(II) generated by in situ electron transfer (Table S2, Supporting Information). Herein, the electron transfer capacity in various reaction systems was revealed by electrochemical tests. In the linear scanning voltammetry (LSV) curves (Figure 5e), the current density of CMC‐GTB/Fe^bm^ reached up to 14.7 µA cm^−2^ at an applied potential of 0.5 V (0.2421 V versus Ag/AgCl), which was significantly better than that of GTB/Fe^bm^ (12.7 µA cm^−2^) and GTB‐Fe (12.2 µA cm^−2^), suggesting that CMC GTB/Fe^bm^ has the best Fe(III) reduction performance.^[^ ^31^ ^]^ A further Tafel test verified the contribution of CMC as a stabilizer to the stability of the confined system (Figure 5f). Cyclic voltammetry (CV) curves confirmed the excellent Fe(III) reduction activity of CMC‐GTB/Fe^bm^ and reflected the enhanced electron‐donating capacity (Figure 5g). This was attributed to the formation of charge transfer channels by confined iron species interacting with the biomass, reducing the charge transfer resistance and mass transfer resistance (Figure 5h).^[^ ^13^ ^]^ Ultimately, the presence of such confined iron species accelerates the extraction of electrons from the biomass, enabling the enrichment and confinement of electrons at the biomass‐solution reaction interface. Benefiting from this strong reduction capacity, CMC‐GTB/Fe^bm^ reduced 100% of Cr(VI). As shown in the deconvolution high‐resolution XPS spectra of Cr 2p, the material tethered Cr is almost exclusively Cr(III) (Figure 5i).^[^ ^7e^ ^]^

It is well known that Cr(VI) exists mainly in the form of HCrO_4_^−^ and CrO_4_^2−^, which are gradually deprotonated with increasing pH. On the other hand, Cr(III) exists mainly in the form of a Cr^3+^ cation, implying that the reduction of Cr(VI) is accompanied by a charge reversal. Zeta potential tests revealed the negative charge property of CMC‐GTB/Fe^bm^ at circumneutral pH (Figure S14, Supporting Information), indicating that the material is favorable for the adsorption and trapping of reduced Cr(III). Therefore, the zeta potential changes of CMC‐GTB/Fe^bm^ in ultrapure water and Cr(VI) solution were examined. As shown in Figure 5j, the rapid positive change in the zeta potential of CMC‐GTB/Fe^bm^ in the Cr(VI) solution was attributed to the coordination trapping of Cr^3+^ ions by the biomass. In addition, since Cr^3+^ has a high charge/radius ratio, its d^3^ configuration maximizes the ligand field stability and promotes the stability of the biomass‐Cr(III) complexes.^[^ ^32^ ^]^ Ultimately, CMC‐GTB/Fe^bm^ stabilizes the tethered fixation of Cr(III) to the biomass surface by coordination, which means that the binding of Cr(III) by the material does not strongly decay with the pH.

In summary, this unique hot‐spot effect‐driven confinement achieves efficient anchoring of iron species on the GTB biomass. The anchored iron allows for efficient electron transfer through active species confinement enhanced metal‐biomass carrier interactions, ultimately leading to 100% Cr(VI) reduction. Meanwhile, Cr(III) is firmly tethered on the GTB biomass due to charge interactions and coordination, rather than in the form of an unstable precipitate of Fe_x_Cr_y_(OH)_3_, as shown in Figure 5k.

### 2.5 Kilogram‐Scale Synthesis and Simulation Application Demonstration

**Figure 6:**
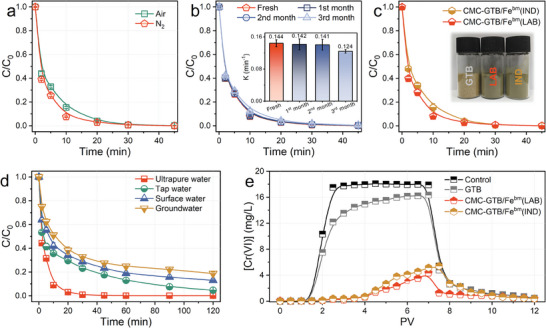
Demonstration of kilogram‐scale synthesis and simulated application of CMC‐GTB/Fe^bm^. a) Effect of headspace gas atmosphere on Cr(VI) removal. b) Effect of different aging times on the reactivity of the material. c) Reactivity of industrially synthesized CMC‐GTB/Fe^bm^(IND). d) Simulated Cr(VI) removal by CMC‐GTB/Fe^bm^(IND) in practical water matrices. e) Removal of injected Cr(VI) by CMC‐GTB/Fe^bm^ loaded media. Reaction conditions: [Cr(VI)]_0_  =  20 mg L^−1^, [Materials]_0_  =  1.0 g L^−1^, pH_0_   6, T = 273 K.

The weak oxidation resistance of the reducing material is an essential challenge that limits the application of iron‐based materials, such as zero‐valent iron.^[^ ^7d^ ^]^ Herein, the high tolerance of CMC‐GTB/Fe^bm^ to dissolved oxygen (DO) in the reaction was demonstrated by adjusting the composition of the headspace gas in the reaction system (**Figure** **6**a). Additionally, the materials were exposed to air aging for several months, and the removal activity of the aged materials for Cr(VI) was tested. As shown in Figure 6b and Figure S15 (Supporting Information), the materials aged for 1–2 months still maintained efficient retention of Cr(VI) removal capacity, with only the material aged for 3 months showing a decrease in kinetics (a 13.9% decrease). A plausible explanation for these phenomena is the antioxidant and capping properties of the plant constituents, which inhibit the oxidation of the material by oxygen from air and solution, thus maintaining the efficient reducing activity of CMC‐GTB/Fe^bm^.^[^ ^33^ ^]^

Owing to the unique advantage of continuous chemical synthesis for easy industrial scale‐up,^[^ ^34^ ^]^ the Cr(VI) removal activity of industrially synthesized CMC‐GTB/Fe^bm^(IND) was evaluated. 5 kg of CMC‐GTB/Fe^bm^(IND) was facilely produced by an industrial ball mill with low synthesis energy consumption of 0.4 kW·h kg^−1^ (Figure S16, Supporting Information). There was no significant difference between the industrially and laboratory‐synthesized CMC‐GTB/Fe^bm^ in terms of apparent and microscopic morphology (Figure 6c; Figure S17, Supporting Information). Moreover, CMC‐GTB/Fe^bm^(IND) also exhibited excellent Cr(VI) removal performance. XPS analysis showed that the surface Fe content of CMC‐GTB/Fe^bm^(IND) was not significantly different from that of the CMC‐GTB/Fe^bm^(LAB) and was similar in composition in characteristic groups, as shown in Figure S18 and Tables S1 and S2 (Supporting Information). These results confirm that the mechanochemically triggered confined coordination strategy is well‐suited for industrial applications.

Under the coexistence of anions and dissolved organic matter, only CO_3_^2−^ leads to a decrease in Cr(VI) removal, attributed to the formation of competitive carbonate‐iron complexes at the reducing active site (Figure S19, Supporting Information). Then, the simulated Cr(VI) removal performance in the actual water matrix showed that the CMC‐GTB/Fe^bm^(IND) was able to maintain a Cr(VI) removal performance of ≈80% in all complex media (Figure 6d). The column breakthrough curves represent the correspondence between effluent Cr(VI) concentration and pore volume (PV), as shown in Figure 6e. The Cr(VI) concentration gradually increased in the control and GTB‐filled groups, and once the Cr(VI) injection was stopped, the Cr(VI) concentration in the effluent rapidly decreased to near zero due to the dilution and replenishment of the background solution. By contrast, in the CMC‐GTB/Fe^bm^ materials‐filled groups, the Cr(VI) concentration appeared only in the last few PVs during Cr(VI) solution injected, and most of the injected Cr(VI) solution was effectively removed by the material. These results indicate that CMC‐GTB/Fe^bm^ possesses strong potential for dynamic Cr(VI) removal in environmental media.

## 3 Conclusion

In this study, to enhance the pH tolerance of green iron‐based materials, a hot spot effect‐triggered confined coordination strategy was proposed to achieve efficient anchoring of iron species on biomass, and the prepared CMC‐GTB/Fe^bm^ was used for the Cr(IV) removal. The results showed that CMC‐GTB/Fe^bm^ achieved 100% reductive detoxification of toxic Cr(VI) anions, and more than 99% removal of Cr(VI) at all circumneutral pH from 5–9, with a maximum removal capacity of up to 180 mg g^−1^. The high detoxification capacity of CMC‐GTB/Fe^bm^ was attributed to the enhancement of interfacial electron transfer caused by the hot spot effect enhanced iron‐phenol coordination reaction. Application experiments confirmed the kilogram‐scale production potential of the proposed strategy and the dynamic Cr(VI) removal performance of the prepared materials in real media. This study developed an industrial scalable mechanochemical‐triggered confined coordination strategy for the preparation of green iron‐based materials, and the proposed concept of open‐space active species confinement provides a reference for Cr(VI) reductive detoxification and tethering at circumneutral pH.

## 4 Experimental Section

### Chemicals and Materials

The manufacturers of the chemical reagents and the recycling procedures of the tea waste are provided in Text S1 (Supporting Information). All reagents are analytically pure and were used without further purification.

### Synthesis of Biomass‐Iron Composites

The synthesis of metal‐biomass composites was achieved through a mechanically triggered confined coordination strategy. Briefly, biomass (1.0 g), iron salts (1.25 mm), and carboxymethyl cellulose (0.1 g) were thoroughly mixed, followed by ball milling to initiate mechanochemical‐triggered iron‐biomass coordination (CMC‐GTB/Fe^bm^). Ball milling was carried out at 400 rpm for 40 min under room temperature (air atmosphere).

### Characterization

Scanning electron microscopy (SEM, TESCAN MIRA‐4, Czech Republic) was utilized to observe the morphology of the materials. Fourier transform infrared spectroscopy (FTIR) was measured on Thermo Fisher Scientific Nicolet iS20 (USA). The crystal phase was analyzed through an X‐ray diffractometer (XRD, Rigaku SmartLab SE, Japan). X‐ray photoelectron spectroscopy (XPS, Thermo Scientific, USA) was applied to analyze the chemical state of iron in the composites. A Zeta potential and Molecular weight analyzer (DLS, Malvern Zetasizer Nano ZS90) was used to detect the zeta potential of the materials. The UV‐Vis spectrometer was used for wavelength scanning from 200–600 nm. Electrochemical tests were performed on an electrochemical workstation (CHI 660D, Chenhua, China) using a three‐electrode system.

### Cr(VI) Removal

All Cr(VI) solutions were freshly prepared and deoxygenated by nitrogen bubbling for 20 min, and then the Cr(VI) removal experiments were conducted in closed reaction bottles. The initial Cr(VI) concentration was set at 20.0 mg L^−1^ with an initial pH of 6.7, and the material dosage was set at 1.0 g L^−1^. The reaction was carried out in a well‐sealed and light‐protected reaction vial of 100 mL, shaken at 220 rpm (25 ± 1 °C). The samples were taken at indicated time points and filtered through 0.22 µm filter membrane. The Cr(VI) concentration was determined by diphenylcarbohydrazide colorimetric method using a UV spectrophotometer at 540 nm with a detection limit of 0.1 mg L^−1^.^[^ ^35^ ^]^ The total Cr concentration was determined by inductively coupled plasma‐mass spectrometry (ICP‐MS, PerkinElmer NexION 300X), with the detection limit of 0.05 µg L^−1^. The Cr(VI) removal efficiency (η, %), removal kinetic constant (k, min^−1^), and removal capacity (Q_e_, mg/g) were calculated as follows:

(1) $$\begin{array}{c} \eta =\frac{C_{0}-C_{t}}{C_{0}}\times 100\; \end{array}$$

(2) $$\begin{array}{c} -\ln(\frac{C}{C_{0}})=k\times t \end{array}$$

(3) $$\begin{array}{c} \;Q_{e}=\frac{C_{0}-C_{t}}{m}\times V\; \end{array}$$

where C_0_ and C_t_ refer to the initial and the real‐time (t, min) concentration of Cr(VI), m (g/L) is the materials dose, and V (mL) is the initial volume of the solution.

## Conflict of Interest

The authors declare no conflict of interest.

## Supporting information



Supporting Information

## Data Availability

The data that support the findings of this study are available from the corresponding author upon reasonable request.
