# Supplementary material for: Mechanochemical‐Triggered Confined Coordination of Iron‐Biomass Composites for Efficient Cr(VI) Reduction Under Circumneutral pH Via Accelerated Electron Extraction
Source: Adv Sci (Weinh). 2025 Feb 24;12(15):2417368. doi: 10.1002/advs.202417368 (PMC12005798; doi:10.1002/advs.202417368)
Supplement: Supplementary file 1 — Supporting Information [file ADVS-12-2417368-s001.docx]

***Supplementary Information*** of

**Mechanochemical-Triggered Confined Coordination of Iron-biomass Composites for Efficient Cr(VI) Reduction Under Circumneutral pH** **via Accelerated Electron Extraction**

Yue Wang ^a^, Hongyu Li ^a^, Hao Liu ^a^, Chengsi Hou ^a^, Zhengwei Zhou ^a^, Shuai Peng ^a^, Zuofeng Chen ^c^, Zhendong Lei ^a^, Deli Wu ^a, b,^ *

*^a^ State Key Laboratory of Pollution Control and Resources Reuse, College of Environmental Science & Engineering, Tongji University, Shanghai 200092, PR China*

*^b^ Shanghai Institute of Pollution Control and Ecological Security, Shanghai 200092, China*

*^c^ School of Chemical Science and Engineering, Tongji University, Shanghai 200092, China*

*Corresponding author: Deli Wu

E-mail: [wudeli@tongji.edu.cn](mailto:wudeli@tongji.edu.cn)

Tel: +86-21-65980872, Fax: +86-21-65983602

**This *Supplementary Information* content:**

[Appendix Supplementary Text (5) S2](#_Toc177655867)

[Appendix Supplementary Table (4) S8](#_Toc177655868)

[Appendix Supplementary Figure (20) S12](#_Toc177655869)

[Reference (19) S31](#_Toc177655870)

## Appendix Supplementary Text

**Text S1 Chemicals and materials**

Potassium dichromate (K_2_Cr_2_O_7_), ferric sulphate (Fe_2_(SO_4_)_3_), ferric chloride (FeCl_3_), sodium chloride (NaCl), sodium hydroxide (NaOH) was analytically pure and purchased from Shanghai Aladdin Biochemical Technology Co. Hydrochloric acid (HCl), acetone (C_3_H_6_O, HPLC grade), Diphenylcarbohydrazide (C_13_H_14_N_4_O) were purchased from Tianjin Solomon Biotechnology Co., Ltd. (Tianjin, China). mZVI and nZVI were purchased from Shanghai Macklin Biochemical Technology Co.

Tea was commercial green tea purchased and collected for use after simulated brewing 5 times (10 min each time) through boiling water at 100°C.

**Text S2 Cr(VI) removal isotherms**

Adsorption isotherm experiments were performed at initial Cr(VI) concentrations of 20, 40, 80, 100, 120, 150, 180, and 200 mg/L, with the CMC-GTB/Fe^bm^ material dosed at 1 g/L and an initial pH of 6.0. Reactions were performed for 24 h to reach Cr(VI) removal equilibrium, and the equilibrium was measured as the Cr(VI) concentration C_e_ (mg/L) in the solution and the equilibrium Cr (VI) removal capacity Q_e_ (mg/g). Subsequently, Langmuir (**Eq. (S1)**) and Freundlich (**Eq. (S2)**) adsorption isotherm models were used to fit the Cr(VI) removal curves:

$$\begin{aligned} Q_{e}=\frac{Q_{m}K_{L}C_{e}}{1+K_{L}C_{e}}\#\left( S1 \right) \end{aligned}$$

$$\begin{aligned} Q_{e}=K_{F}C_{e}^{1/n}\#\left( S2 \right) \end{aligned}$$

where K_L_ is the equilibrium adsorption constant, which is related to the affinity of the binding site (L/mg). K_F_ and 1/n are constants related to the adsorption capacity and affinity between the Cr(V) and the CMC-GTB/Fe^bm^ adsorbent, respectively.

**Text S3 Phytotoxicity**

***Plant, grouping and exposure experiments***

Wheat, an important cash crop, was selected as the model plant in the toxicity experiments. The 20 mg/L Cr(VI) and ultrapure water groups were designated as the positive and negative control groups, while GTB, GTB/Fe^bm^, and CMC-GTB/Fe^bm^-treated Cr(VI) solutions were set up as the experimental groups. Forty randomly selected wheat seeds from each group were incubated with 15 mL of the specified solutions using filter paper as a carrier. After 7 d of exposure treatment, the wheat germination rate, plant biomass (root biomass and shoot biomass), and chlorophyll a content was determined.

***Plant biomass***

After 7 d of exposure experiment, wheat shoot and root were separated and dry weight of biomass after freeze drying was recorded.

***Chlorophyll a content measurement***

Wheat malt after growing for 7 d was collected and weighed (m). A total of 4 mL of 95% ethanol was added to the crushed tissue homogenate and ultrasonicated to mix thoroughly. The ethanol solution was dark treated at 4°C for 24 h to extract chlorophyll from the malt. Subsequently, the chlorophyll-containing supernatant was obtained through 10000 rpm centrifugation at 4°C for 15 min, and the absorbance of the solution was measured at 665 nm (A_665 nm_) and 649 nm (A_649 nm_) using UV-vis and the chlorophyll a (Chl_a_) content was calculated.

$$\begin{aligned} \text{Chl}_{\text{a}}\text{ }\left( \text{μg}\text{/mg} \right)\text{ }\text{=}\text{ }\frac{\left( \text{13.95×}\text{A}_{\text{665 nm}}\text{-}\text{6.88×}\text{A}_{\text{649 nm}} \right)}{\text{m}}\text{ }\#\left( \text{S1} \right) \end{aligned}$$

***Statistical analysis***

Data for phytotoxicity analyses were presented as mean ± standard error (SE), and all treatment groups were replicated three times. One-way analysis of variance (ANOVA) was performed using IBM SPSS 25.0 (Armonk, USA), whereas *P* < 0.05 was considered as significant difference between groups.

**Text S4 Electrochemical analysis**

Electrochemical measurements were recorded on a CHI 660D electrochemical workstation. The material (8 mg) was dispersed into a mixed solution containing ethanol (980 µL) and Nafion (20 µL) and mixed well. The working electrode was prepared by dropping 10 μL of the dispersed solution onto a 3 mm diameter glassy carbon electrode (GCE). Pt electrode and saturated Ag/AgCl electrode were used as counter electrode and reference electrode, respectively. Prior to electrochemical testing, the electrolyte solution (3.5%_wt_ NaCl solution, pH = 1) was bubbled with nitrogen to remove dissolved oxygen.

The measured potential to the reversible hydrogen electrode (RHE) was calibrated with the following equation [1]:

$$\begin{aligned} E_{RHE}=E_{Ag/AgCl}+0.059\times pH+0.1989 V \#\left( S2 \right) \end{aligned}$$

**Text S5 Column experiment**

To simulate the dynamic remediation of Cr(VI) by CMC-GTB/Fe^bm^, column breakthrough experiments were performed in a quartz sand/material mix-filled PMMA column (**Fig. S20**). The porosity of the quartz sand (20-50 mesh) was 0.43 and the calculated pore volume (PV) of the column was 30 ml. Briefly, GTB, CMC-GTB/Fe^bm^, industrially manufactured CMC-GTB/Fe^bm^ were mixed with quartz sand at a mass ratio of 1:100. A column of quartz sand without mixing any material was taken as the control. The filled PMMA column was placed vertically, and 5 PV of background electrolyte solution (10 mM NaCl, pH 7) was passed through the column from the bottom up first, followed by 5 PV of Cr(VI) solution (10 mg/L, pH 6.7) pumped at a flow rate of 2 mL/min, and finally 5 PV ultrapure water was pumped for washing. The effluent samples were collected at the indicated times and the concentration of Cr(VI) was measured.

##

## Appendix Supplementary Table

**Table S1.** Semi-quantitative results for the elements C, O, Fe and Cr obtained through XPS analyses.

|  | GTB-Fe | CMC-GTB/Fe^bm^ | CMC-GTB/Fe^bm^(IND) | CMC-GTB/Fe^bm^(Cr) |
| --- | --- | --- | --- | --- |
| C 1s | 76.28 | 69.81 | 70.17 | 71.13 |
| O 1s | 23.25 | 28.88 | 28.44 | 27.81 |
| Fe 2p | 0.46 | 1.31 | 1.38 | 0.66 |
| Cr 2p | -- | -- | -- | 0.39 |

**Table S2.** The positions and contents of the chemical groups obtained from XPS high-resolution spectra analysis.

| Element | B.E. (eV) | Group | GTB-Fe | CMC-GTB/Fe^bm^ | CMC-GTB/Fe^bm^(IND) | CMC-GTB/Fe^bm^(Cr) |
| --- | --- | --- | --- | --- | --- | --- |
| C 1s | 284.18-284.28 | C-C/C=C | 67.74 | 63.97 | 69.22 | 64.03 |
|  | 285.65-285.80 | C=O | 27.48 | 29.95 | 25.64 | 30.03 |
|  | 288.00-288.11 | -COOH | 4.78 | 6.08 | 5.14 | 5.94 |
| O 1s | 531.00-531.20 | C-O | 26.26 | 38.56 | 40.12 | 24.90 |
|  | 531.77-532.05 | C-O-Fe | 46.47 | 38.96 | 41.67 | 39.25 |
|  | 532.60-532.79 | C-O-C | 27.27 | 22.48 | 18.21 | 35.85 |
| Fe 2p | 710.16-710.56, 723.45-723.80 | Fe(II) | 52.00 | 32.48 | 39.81 | 59.34 |
|  | 712.23-712.91, 725.45-725.80 | Fe(III) | 48.00 | 67.52 | 60.19 | 40.66 |

**Table S3.** Isotherm fitting for Cr(VI) removal by CMC-GTB/Fe^bm^ materials.

| Models | Langmuir | | | Freundlich | | |
| --- | --- | --- | --- | --- | --- | --- |
| Parameters | K_L_  (L/mg) | Q_m_  (mg/g) | R^2^ | K_F_  [(mg/g)·(mg/L)^-1/n^] | 1/n | R^2^ |
| CMC-GTB/Fe^bm^ | 0.35 | 180.2 | 0.976 | 60.6 | 0.32 | 0.979 |

**Table S4.** Comparison of the Cr(VI) reduction removal performance of iron-based materials reported in the historical literature.

| Mat. | Mechanism | pH_0_ | [Mat.]_0_ | Q_max_ | *Ref.* |
| --- | --- | --- | --- | --- | --- |
|  |  |  | g/L | mg/g |  |
| nZVI | Adsorption & reduction | 6.5 | 1.0 | 7.8 | [3] |
| mZVI | Adsorption & reduction | 6.2 | 0.1 | 0.87 | [4] |
| Fe@PC | Adsorption & reduction | 1.0 | 2.0 | 10.0 | [5] |
| TS-BZVI | Adsorption & reduction | 5.7 | 1.3 | 11.0 | [6] |
| mZVI/AC | Adsorption & reduction | 3.0 | 1.0 | 14.4 | [7] |
| Fe^0^-Biochar | Adsorption & reduction | 5.5 | 2.0 | 19.6 | [8] |
| PA-ZVI^bm^ | Adsorption & reduction | 5.8 | 3.0 | 22.1 | [10] |
| nZVI/EaHC | Adsorption & reduction | 2.0 | 0.1 | 49.9 | [11] |
| S-mZVI/SGB | Capture & reduction | 5.7 | 1.0 | 70.2 | [12] |
| nZVI-BC | Adsorption & reduction | 6.0 | 1.0 | 86.1 | [13] |
| Fe-NPs (Green) | Adsorption & reduction | 4.0 | 1.4 | 20.5 | [9] |
| YL-FeNPs (Pear) | Reduction & coprecipitation | 5.0 | 0.4 | 44.0 | [14] |
| P-nZVI (*Palm Petiole*) | Adsorption & reduction | 5.0 | 1.0 | 44.5 | [15] |
| KP-FeNPs (Pear) | Adsorption & reduction | 5.0 | 0.4 | 46.6 | [16] |
| S/YP-INPs@CB | Reduction & coprecipitation | 5.0 | 0.4 | 79.7 | [18] |
| Fe-NPs (*Eucalyptus*) | Adsorption & reduction | 4.0 | 0.5 | 144.9 | [17] |
| CMC-GTB/Fe^bm^ | Reduction & fixation | 6.0 | 1.0 | 180.0 | **This work** |

## Appendix Supplementary Figure


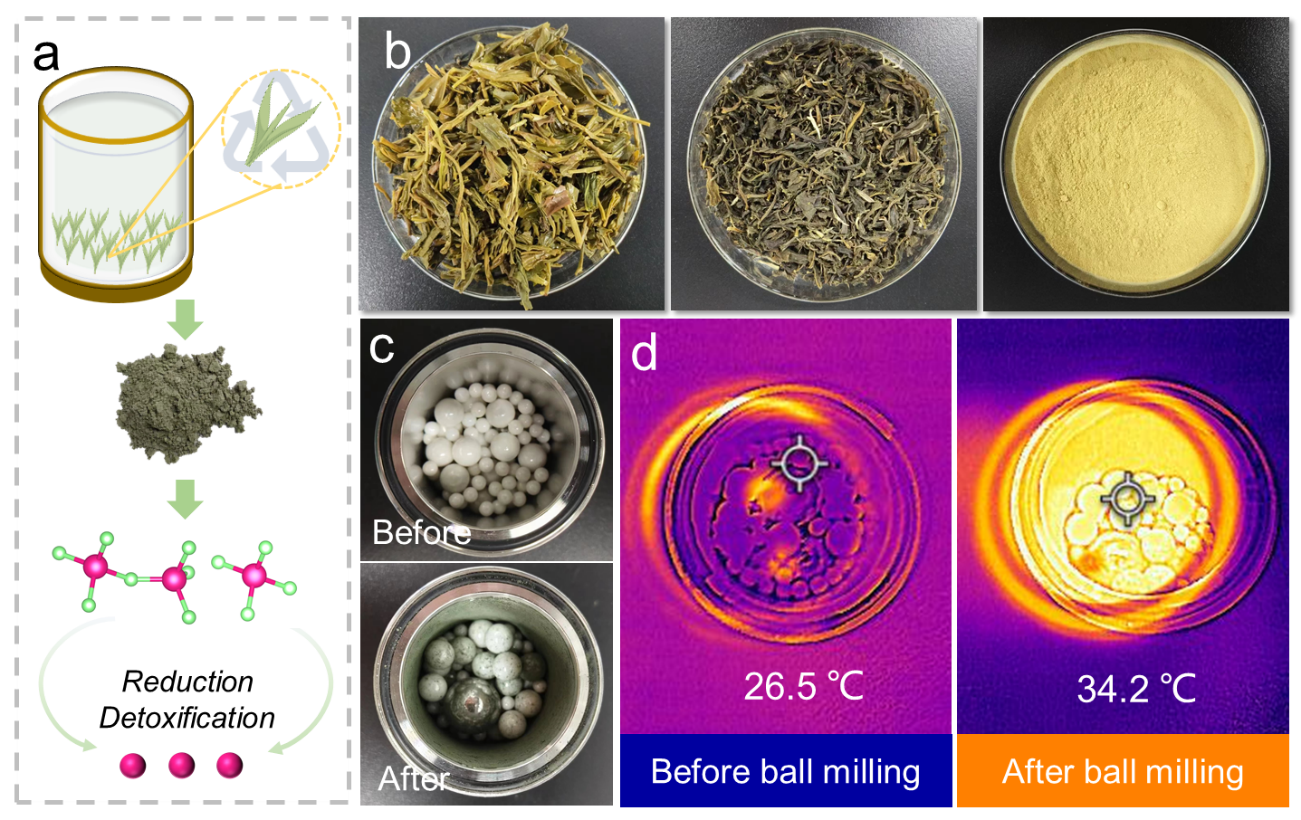


**Fig. S1.** **Utilization of recycled waste tea biomass for efficient Cr(VI) reduction in aqueous solution.** (a) Recycling of tea biomass for Cr(VI) reduction and detoxification. (b) Pre-treatment of waste tea. (c) Optical and (d) thermographic images of the ball-milled tank before and after reaction.


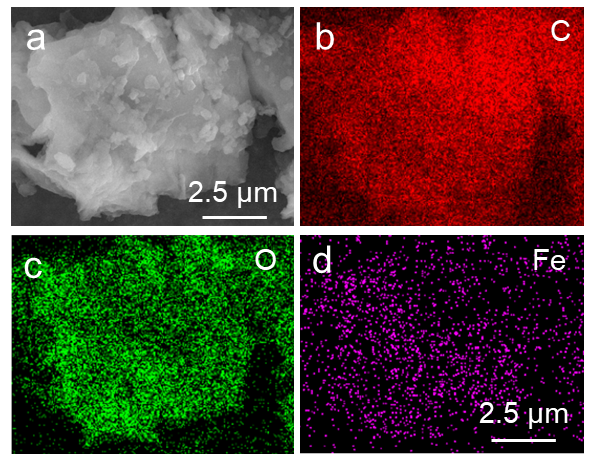


**Fig. S2.** SEM-Mapping images of biomass-iron isolated from a physically mixed solution of biomass and iron salt.


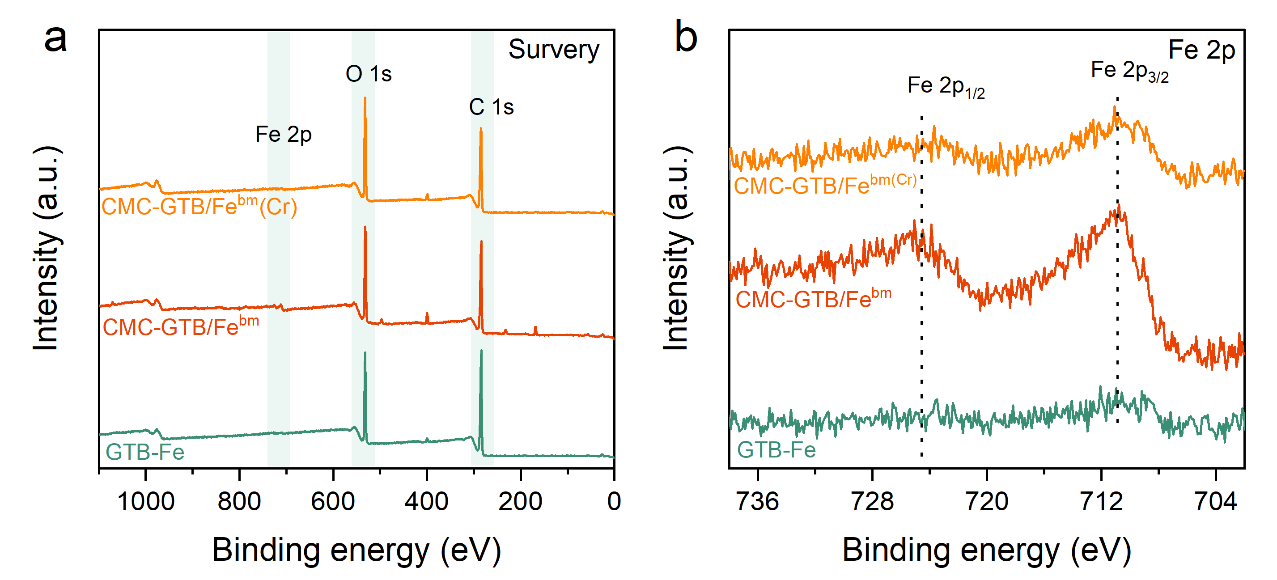


**Fig. S3.** XPS spectra of different materials: (a) Survey spectra and (b) high-resolution spectra of Fe 2p.


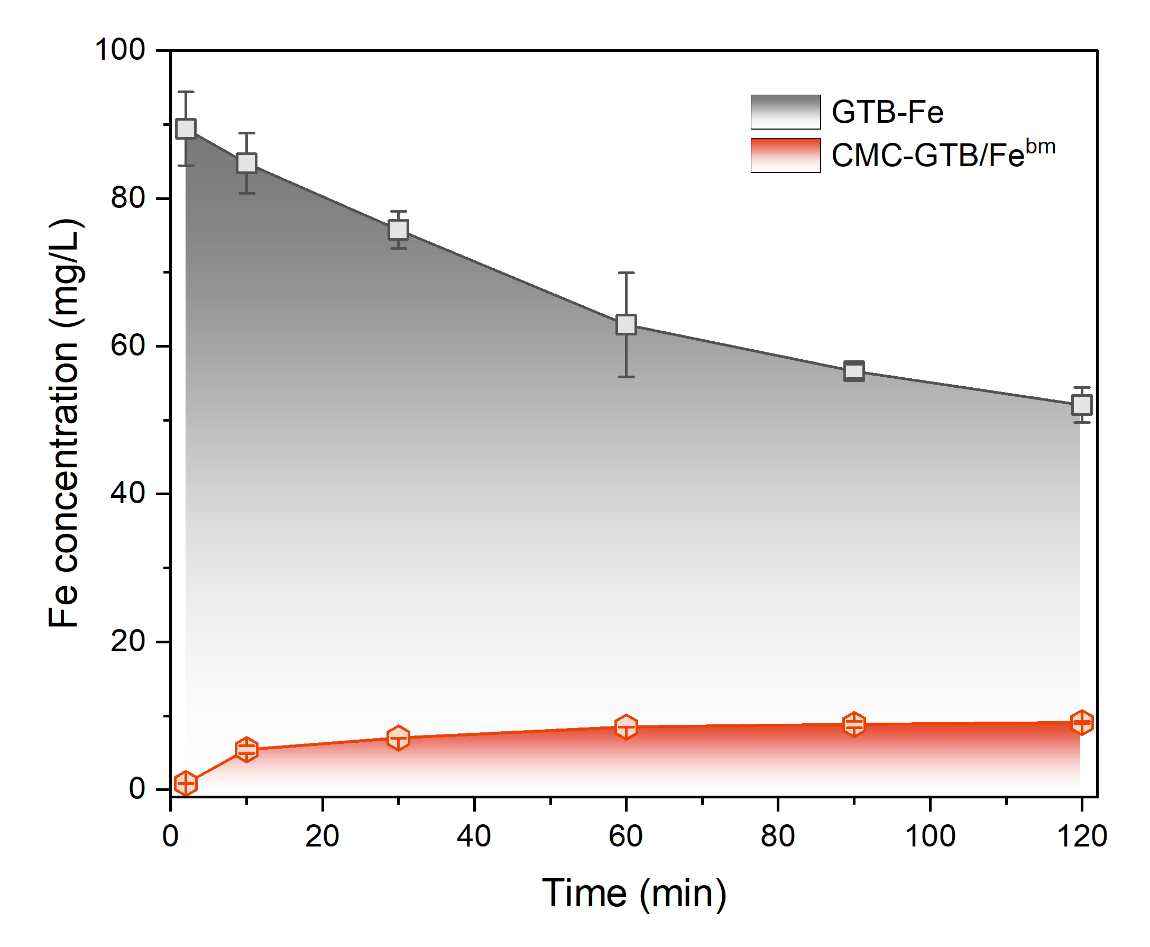


**Fig. S4.** Iron release of GTB-Fe and CMC-GTB/Fe^bm^. Reaction conditions: [Materials]_0_ = 1.0 g/L, pH unadjusted, T = 273 K.


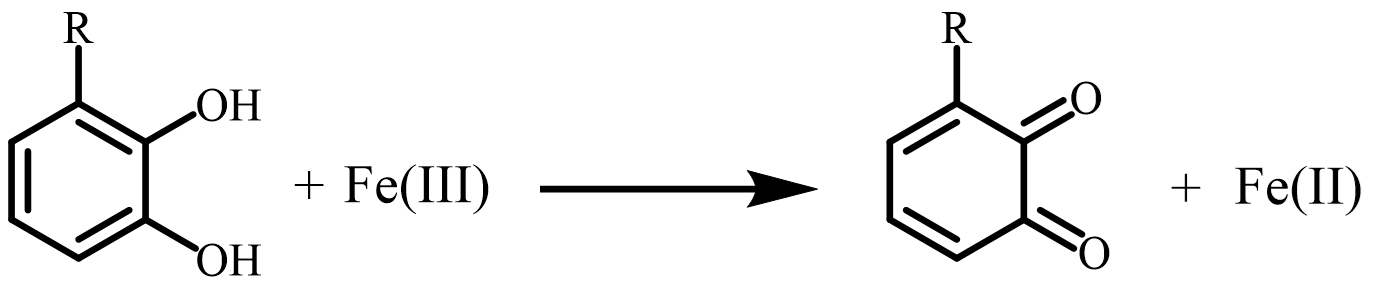


**Fig. S5.** Phenols reduce Fe(III) to Fe(II) by single electron transfer and generate quinone.


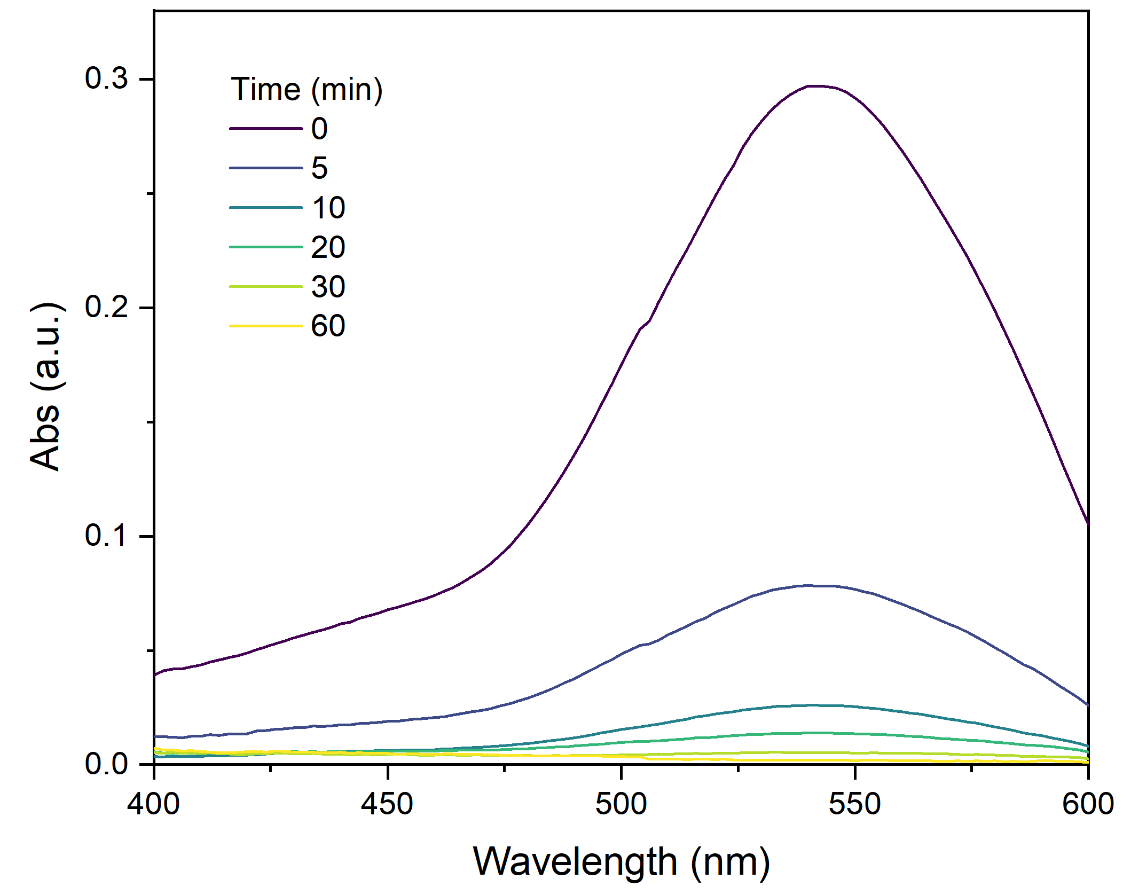


**Fig. S6.** Representative UV-vis spectra of Cr(VI) removal by CMC-GTB/Febm material. Reaction conditions: [Cr(VI)]_0_ = 20 mg/L, [Materials]_0_ = 1.0 g/L, pH_0_ = 6, T = 273 K.

**Fig. S7.** Long-term removal of Cr(VI) by GTB and GTB^bm^. Reaction conditions: [Cr(VI)]_0_ = 20 mg/L, [Materials]_0_ = 1.0 g/L, pH_0_ = 6, T = 273 K.

**Fig. S8.** Generalization of the “mechanochemical triggered iron-biomass confined coordination” strategy for various precursor parameters. (a) Comparison of ferrous sulfate (CMC-GTB/Fe(II)^bm^) and iron sulfate (CMC-GTB/Fe(II)^bm^) as the iron precursor. (b) Comparison of different biomass precursors, tea leaves (GTB), coffee grounds (CFB) and orange peels (OPB). Reaction conditions: [Cr(VI)]_0_ = 20 mg/L, [Materials]_0_ = 1.0 g/L, pH_0_ = 6, T = 273 K.

***Supplementary Note:*** The universality evaluation showed that there was no significant difference between CMC-GTB/Fe(II)^bm^ and CMC-GTB/Fe(III)^bm^ in Cr(VI) reduction kinetics, and the high stability of trivalent iron salts offers further potential for application (**Fig. S8a**). The comparison of the Cr(VI) removal kinetics of the materials prepared from various biomass precursors showed that the Cr(VI) removal performance of “CMC-GTB/Fe^bm^ > CMC-CFB/Fe^bm^ > CMC-OPB/Fe^bm^”, as shown in **Fig. S8b**.


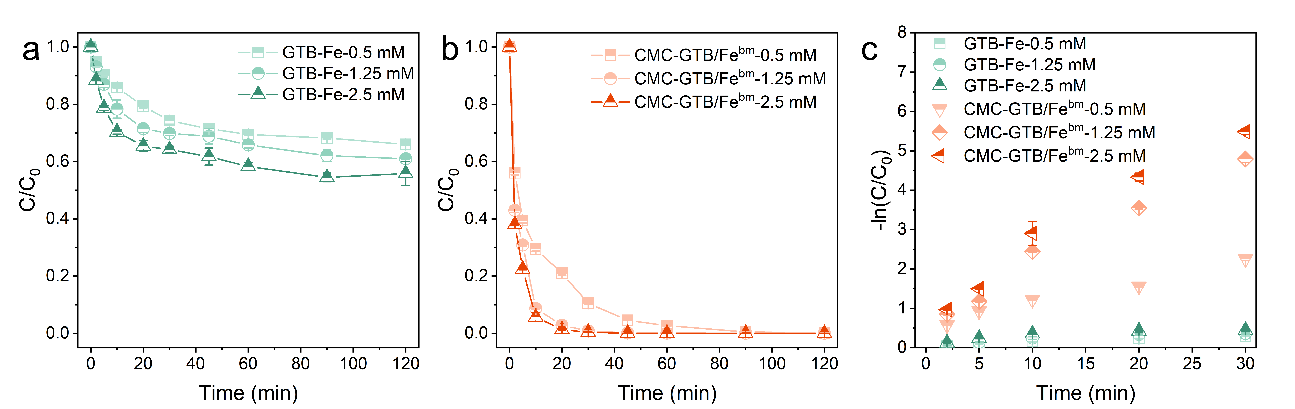


**Fig. S9.** Cr(VI) removal curves of (a) GTB-Fe and (b) CMC-GTB/Fe^bm^ systems prepared with different iron salt ratios. (c) Cr(VI) removal kinetics fitting. Reaction conditions: [Cr(VI)]_0_ = 20 mg/L, [Materials]_0_ = 1.0 g/L, pH_0_ = 6, T = 273 K.

**Fig. S10.** Cr(VI) removal kinetics fitting for each reaction system. Reaction conditions: [Cr(VI)]_0_ = 20 mg/L, [Materials]_0_ = 1.0 g/L, pH_0_ = 6, T = 273 K.


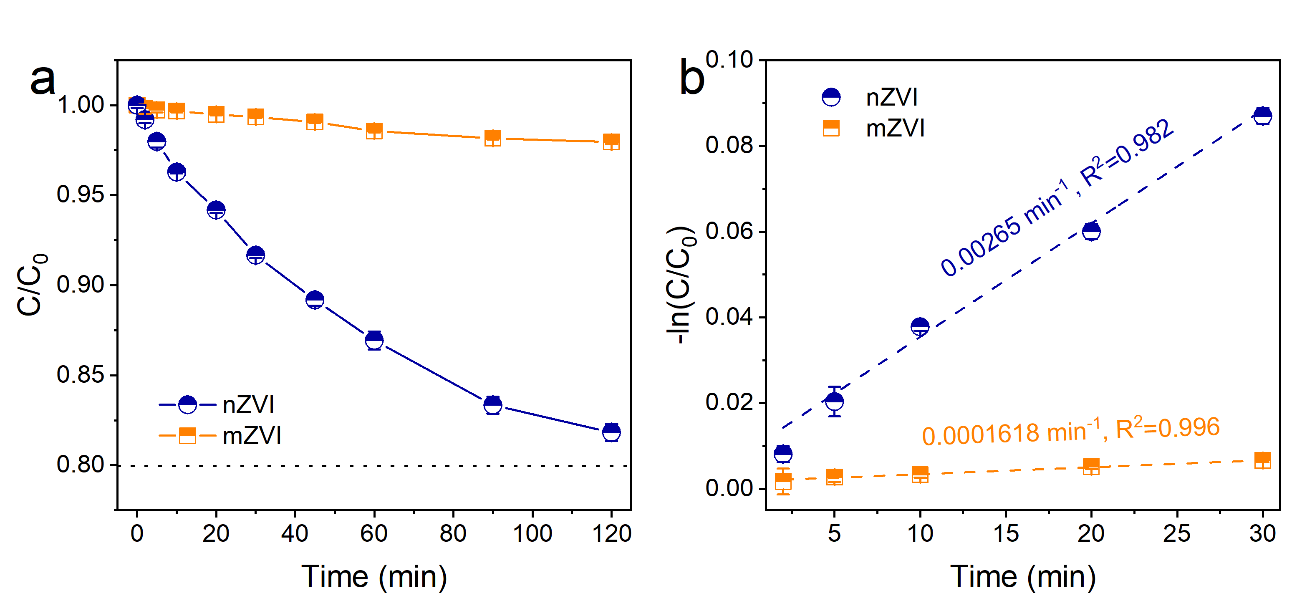


**Fig. S11.** (a) Removal curves and (b) kinetic fitting for Cr(VI) removal by nZVI and mZVI. Reaction conditions: [Cr(VI)]_0_ = 20 mg/L, [Materials]_0_ = 1.0 g/L, pH_0_ = 6, T = 273 K.


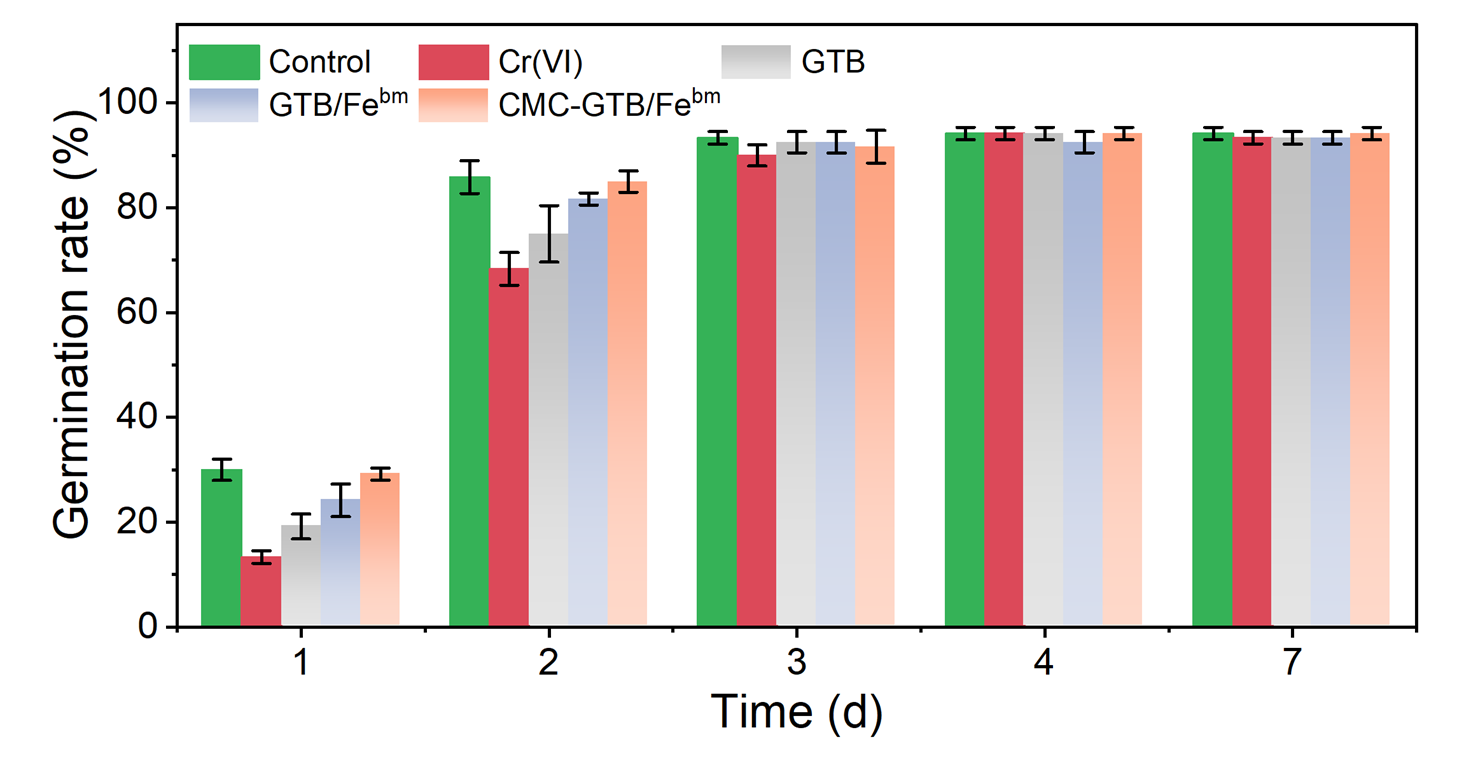


**Fig. S12.** Temporal variation in germination rates of wheat (*Triticum aestivum L*).


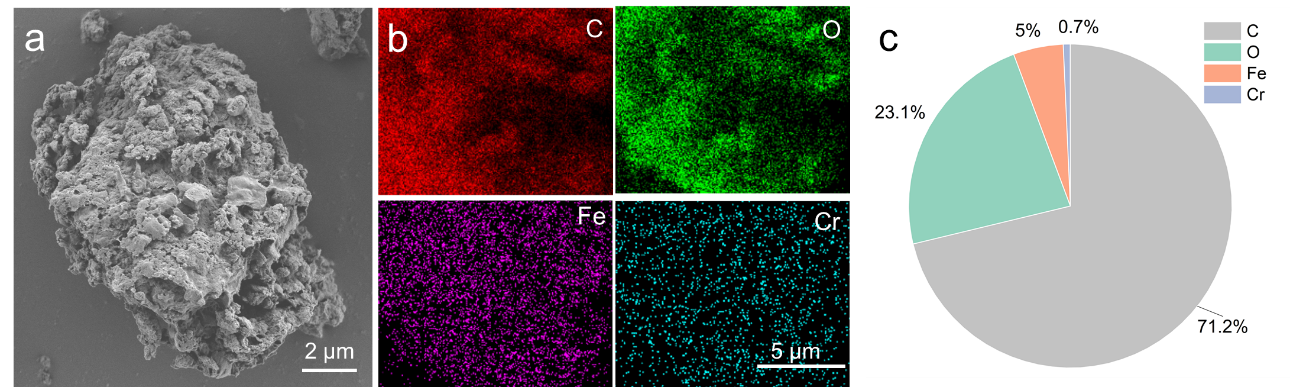


**Fig. S13.** SEM-EDS images of CMC-GTB/Fe^bm^ after Cr(VI) removal. (a) SEM image, (b) EDS-mapping images and (c) elements content of CMC-GTB/Fe^bm^(Cr).

***Supplementary Note:*** The surface of the separated CMC-GTB/Fe^bm^ material was rougher after the Cr(VI) removal test (**Fig. S13a**), attributed to the dissolution and surface aggregation of the biomass constituents. The EDS-mapping results showed a homogeneous distribution of Cr, as shown in **Fig. S13b**. Semi-quantitative analyses of the elements showed a small loss of Fe, indicating that the material was still able to maintain an effective Fe loading during the reaction (**Fig. S13c**), whilst an increase in the surface C content confirmed the aggregation of organic matter on the surface.

**Fig. S14.** Zeta potential of CMC-GTB/Fe^bm^ at pH 5-9.

**Fig. S15.** Kinetic fitting of Cr(VI) removal curves by aged CMC-GTB/Fe^bm^. Reaction conditions: [Cr(VI)]_0_ = 20 mg/L, [Materials]_0_ = 1.0 g/L, pH_0_ = 6, T = 273 K.


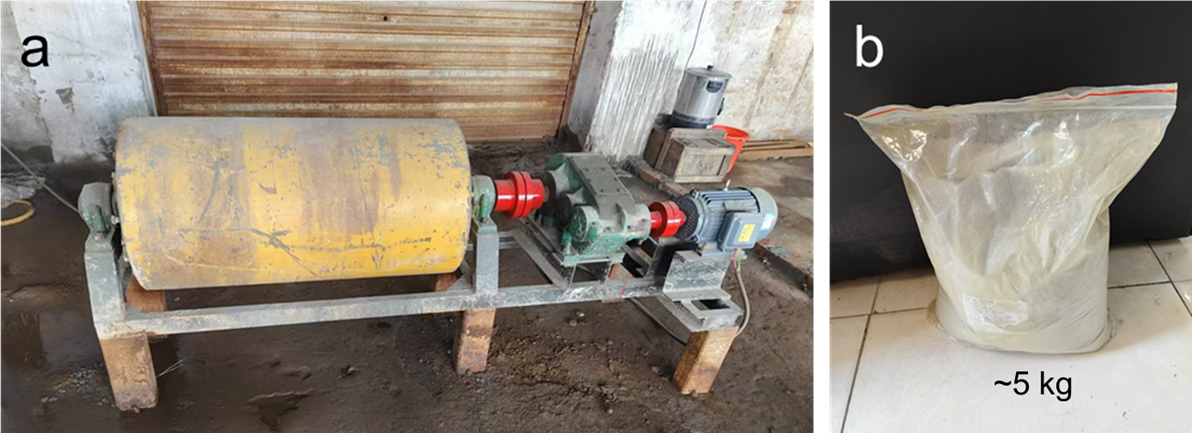


**Fig. S16.** Industrial synthesis of 5 kg of CMC-GTB/Fe^bm^(IND). (a) The used industrial ball miller. (b) Picture of 5 kg of CMC-GTB/Fe^bm^(IND) material.


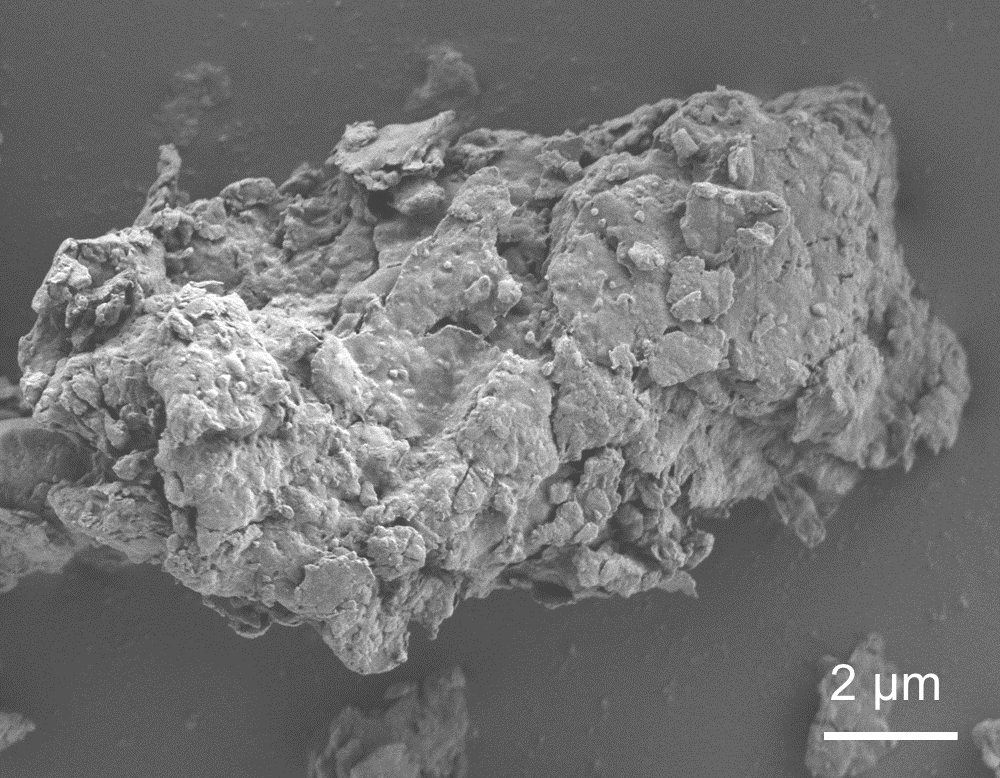


**Fig. S17.** SEM image of as-prepared CMC-GTB/Fe^bm^(IND).


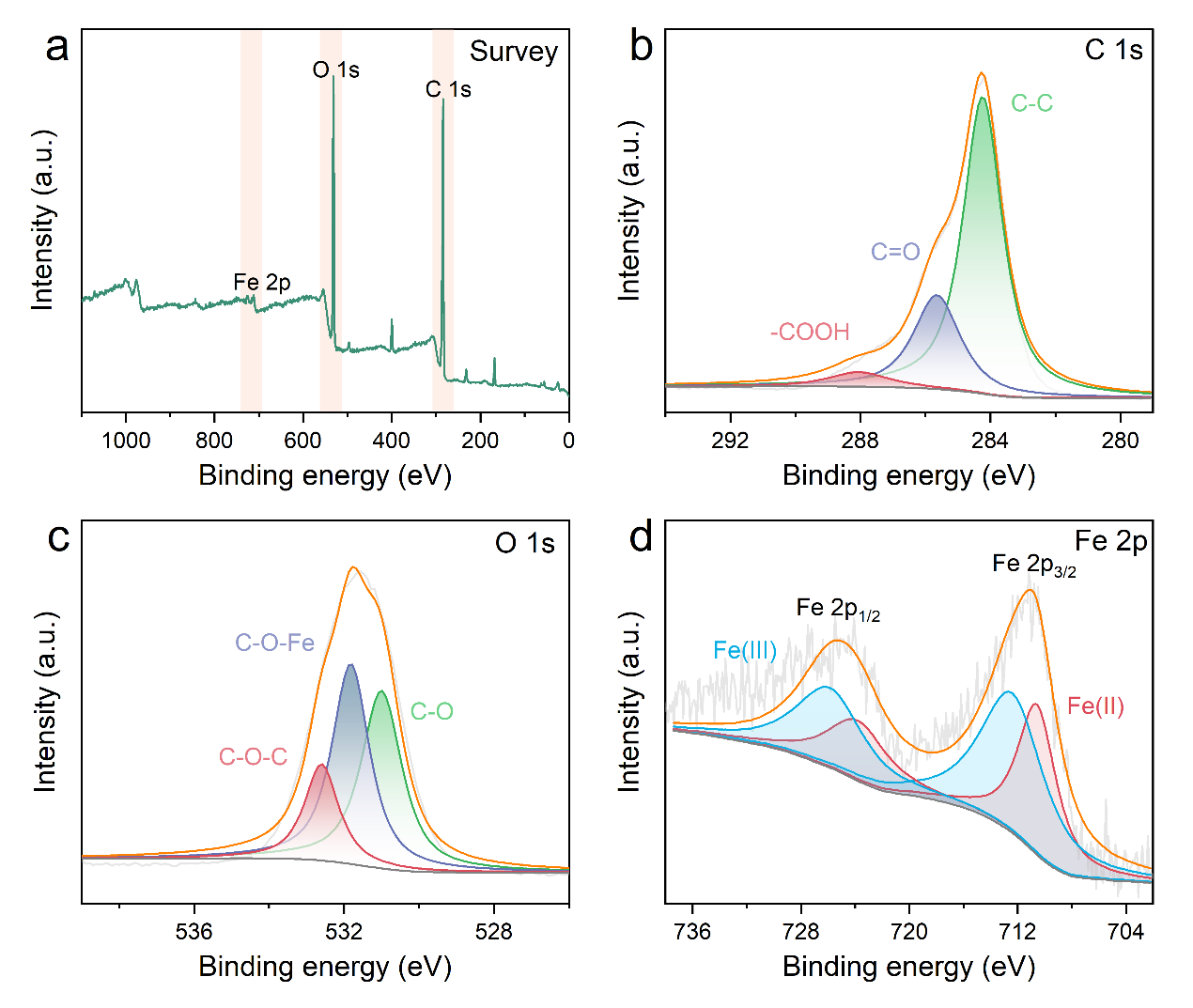


**Fig. S18.** XPS spectra of CMC-GTB/Fe^bm^(IND). (a) Survey spectra and high-resolution spectra of (b) C 1s, (c) O 1s and (d) Fe 2p.


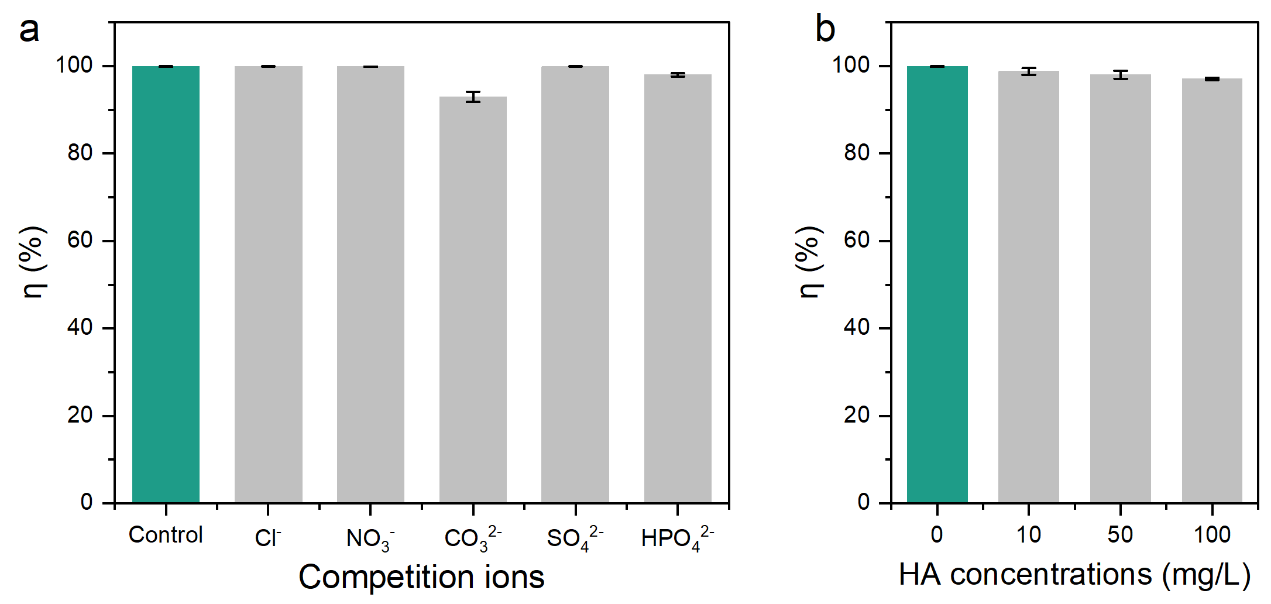


**Fig. S19.** The role of competing ions and dissolved organic matter. (a) Effect of competing ions (10 mg/L) on Cr(VI) removal. (b) Effect of gradient concentration humic acid (HA) dosing on Cr(VI) removal. Reaction conditions: [Cr(VI)]_0_ = 20 mg/L, [CMC-GTB/Fe^bm^(IND)]_0_ = 1.0 g/L, pH_0_ = 6, T = 273 K.

***Supplementary Note:*** Inhibition experiments showed that the competing ion CO_3_^2-^ had the strongest inhibition of Cr(VI) removal, attributed to the competition for the active site of the Cr(VI) reduction reaction, leading to the generation of inert Fe-carbonate complexes [19]. In addition, HA showed almost no inhibition of Cr(VI) removal, similar to the previous report [20].


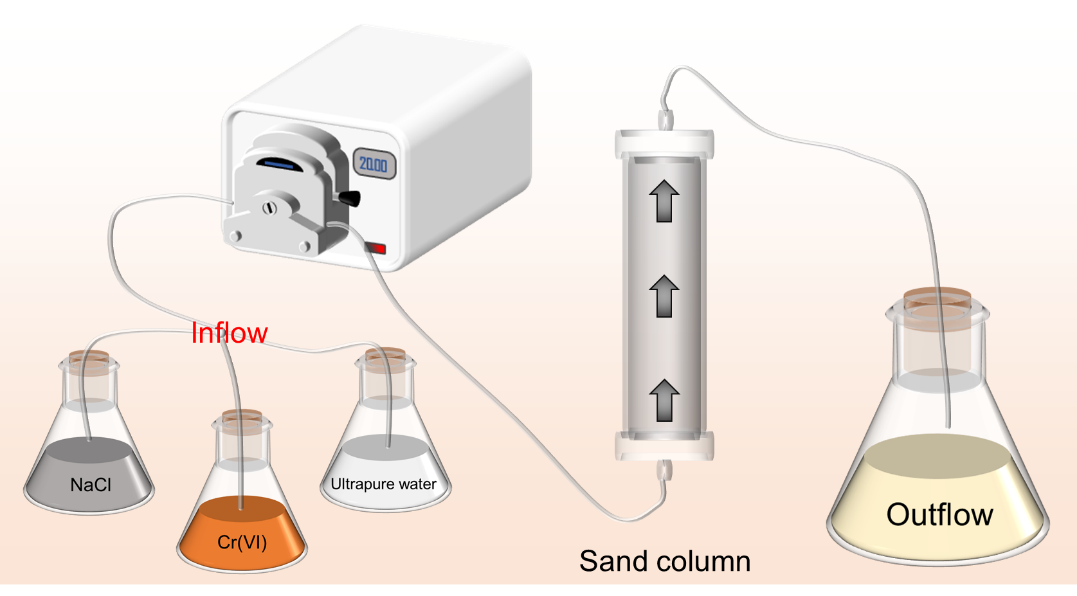


**Fig. S20.** Diagram of the column experimental device.

## Reference

[1] C. Chen, Z. Fu, F. Qi, Y. Chen, G. Meng, Z. Chang, F. Kong, L. Zhu, H. Tian, H. Huang, X. Cui, J. Shi, *Angew. Chem. Int. Ed.* **2022**, *61* (32), e202207226, <https://doi.org/https://doi.org/10.1002/anie.202207226>.

[2] M. Cai, Y. Liu, K. Dong, X. Chen, S. Li, *Chin. J. Catal.* **2023**, *52*, 239, <https://doi.org/https://doi.org/10.1016/S1872-2067(23)64496-1>.

[3] Z. Ai, Y. Cheng, L. Zhang, J. Qiu, *Environ. Sci. Technol.* **2008**, *42* (18), 6955, <https://doi.org/10.1021/es800962m>.

[4] Y. Hu, X. Peng, Z. Ai, F. Jia, L. Zhang, *Environ. Sci. Technol.* **2019**, *53* (14), 8333, <https://doi.org/10.1021/acs.est.9b01999>.

[5] L. Zhuang, Q. Li, J. Chen, B. Ma, S. Chen, *Chem. Eng. J.* **2014**, *253*, 24, <https://doi.org/https://doi.org/10.1016/j.cej.2014.05.038>.

[6] X. Wang, S. Yuan, J. Kong, C. Chen, C. Yu, L. Huang, H. Sun, X. Peng, Y. Hu, *J. Hazard. Mater.* **2024**, *473*, 134668, <https://doi.org/https://doi.org/10.1016/j.jhazmat.2024.134668>.

[7] W. Wang, B. Hu, C. Wang, Z. Liang, F. Cui, Z. Zhao, C. Yang, *Chem. Eng. J.* **2020**, *389*, 122633, <https://doi.org/https://doi.org/10.1016/j.cej.2019.122633>.

[8] K. Wang, Y. Sun, J. Tang, J. He, H. Sun, *Chemosphere* **2020**, *241*, 125044, <https://doi.org/https://doi.org/10.1016/j.chemosphere.2019.125044>.

[9] X. Jin, Y. Liu, J. Tan, G. Owens, Z. Chen, *J. Cleaner Prod.* **2018**, *176*, 929, <https://doi.org/https://doi.org/10.1016/j.jclepro.2017.12.026>.

[10] M. Zhuo, D. Zheng, G. Lu, G. Zhang, J. Chen, Y. Song, *J. Hazard. Mater.* **2025**, *483*, 136670, <https://doi.org/https://doi.org/10.1016/j.jhazmat.2024.136670>.

[11] Y. Ma, N. Lu, S. Yan, H. Wang, X. Cao, T. Feike, J. Guan, *Sep. Purif. Technol.* **2025**, *358*, 130423, <https://doi.org/https://doi.org/10.1016/j.seppur.2024.130423>.

[12] Y. Wang, Z. Liu, W. Huang, J. Lu, S. Luo, B. Czech, T. Li, H. Wang, *Carbon Research* **2023**, *2* (1), 11, <https://doi.org/10.1007/s44246-023-00044-6>.

[13] Y. Wei, R. Chu, Q. Zhang, M. Usman, F. U. Haider, L. Cai, *RSC Adv.* **2022**, *12* (41), 26953, <https://doi.org/https://doi.org/10.1039/d2ra04650d>.

[14] K. Rong, J. Wang, X. Li, Z. Zhang, Q. Yang, C. Shan, T. Wu, J. Liu, *Biomass Convers. Biorefin.* **2024**, *14* (3), 4355, <https://doi.org/10.1007/s13399-022-02464-7>.

[15] D. Tesnim, B. A. Hédi, D. Ridha, A. Cid-Samamed, *Environ. Sci. Pollut. Res.* **2024**, *31* (31), 44272, <https://doi.org/10.1007/s11356-024-34092-1>.

[16] K. Rong, J. Wang, Z. Zhang, J. Zhang, *Ecol. Eng.* **2020**, *149*, 105793, <https://doi.org/https://doi.org/10.1016/j.ecoleng.2020.105793>.

[17] X. Weng, X. Jin, J. Lin, R. Naidu, Z. Chen, *Ecol. Eng.* **2016**, *97*, 32, <https://doi.org/https://doi.org/10.1016/j.ecoleng.2016.08.003>.

[18] K. Rong, X. Li, Q. Yang, Z. Liu, Q. Yao, Z. Zhang, R. Li, L. Zhao, H. Zheng, *Appl. Surf. Sci.* **2024**, *670*, 160729, <https://doi.org/https://doi.org/10.1016/j.apsusc.2024.160729>.

[19] C. L. Caldeira, V. S. T. Ciminelli, K. Osseo-Asare, *Geochim. Cosmochim. Acta* **2010**, *74* (6), 1777, <https://doi.org/https://doi.org/10.1016/j.gca.2009.12.014>.

[20] T. Liu, P. Rao, I. M. C. Lo, *Sci. Total Environ.* **2009**, *407* (10), 3407, <https://doi.org/https://doi.org/10.1016/j.scitotenv.2009.01.043>.
